# Altered functional brain dynamics in chromosome 22q11.2 deletion syndrome during facial affect processing

**DOI:** 10.1038/s41380-021-01302-y

**Published:** 2021-10-22

**Authors:** Eli J. Cornblath, Arun Mahadevan, Xiaosong He, Kosha Ruparel, David M. Lydon-Staley, Tyler M. Moore, Ruben C. Gur, Elaine H. Zackai, Beverly Emanuel, Donna M. McDonald-McGinn, Daniel H. Wolf, Theodore D. Satterthwaite, David R. Roalf, Raquel E. Gur, Dani S. Bassett

**Affiliations:** 1 Department of Neuroscience, Perelman School of Medicine, Philadelphia, PA USA; 2 Department of Bioengineering, School of Engineering & Applied Science, Philadelphia, PA USA; 3 Department of Psychiatry, Perelman School of Medicine, Philadelphia, PA USA; 4 Department of Neurology, Perelman School of Medicine, Philadelphia, PA USA; 5 Department of Radiology, Perelman School of Medicine, Philadelphia, PA USA; 6 22q and You and Clinical Genetics Centers, Children’s Hospital of Philadelphia, Philadelphia, PA USA; 7 Division of Human Genetics, Department of Pediatrics, Children’s Hospital of Philadelphia, Perelman School of Medicine, University of Pennsylvania, Philadelphia, PA USA; 8 Department of Physics & Astronomy, College of Arts & Sciences, Philadelphia, PA USA; 9 Department of Electrical & Systems Engineering, School of Engineering & Applied Science, Philadelphia, PA USA; 10 Santa Fe Institute, Santa Fe, NM USA; 11 Department of Biostatistics, Epidemiology, & Informatics, Perelman School of Medicine, Philadelphia, PA USA

**Keywords:** Schizophrenia, Neuroscience, Psychology

## Abstract

Chromosome 22q11.2 deletion syndrome (22q11.2DS) is a multisystem disorder associated with multiple congenital anomalies, variable medical features, and neurodevelopmental differences resulting in diverse psychiatric phenotypes, including marked deficits in facial memory and social cognition. Neuroimaging in individuals with 22q11.2DS has revealed differences relative to matched controls in BOLD fMRI activation during facial affect processing tasks. However, time-varying interactions between brain areas during facial affect processing have not yet been studied with BOLD fMRI in 22q11.2DS. We applied constrained principal component analysis to identify temporally overlapping brain activation patterns from BOLD fMRI data acquired during an emotion identification task from 58 individuals with 22q11.2DS and 58 age-, race-, and sex-matched healthy controls. Delayed frontal-motor feedback signals were diminished in individuals with 22q11.2DS, as were delayed emotional memory signals engaging amygdala, hippocampus, and entorhinal cortex. Early task-related engagement of motor and visual cortices and salience-related insular activation were relatively preserved in 22q11.2DS. Insular activation was associated with task performance within the 22q11.2DS sample. Differences in cortical surface area, but not cortical thickness, showed spatial alignment with an activation pattern associated with face processing. These findings suggest that relative to matched controls, primary visual processing and insular function are relatively intact in individuals with 22q11.22DS, while motor feedback, face processing, and emotional memory processes are more affected. Such insights may help inform potential interventional targets and enhance the specificity of neuroimaging indices of cognitive dysfunction in 22q11.2DS.

## Introduction

Chromosome 22q11.2 deletion syndrome (22q11.2DS) is a genetic neurodevelopmental disorder characterized by a submicroscopic deletion of the long arm of chromosome 22q [1], which causes a heterogeneous mix of cardiac, endocrine, palatal, immune, gastrointestinal, genitourinary, skeletal, and psychiatric abnormalities [1]. 22q11.2DS is one of the strongest genetic risk factors for psychosis, with over 25% prevalence of psychosis-spectrum symptoms in affected adults [2, 3], alongside comorbid autism spectrum, attention-deficit, anxiety, and mood symptoms [3, 4].

Despite the range of neuropsychiatric symptoms in 22q11.2DS, this population is often studied in attempts to identify early structural and functional neuroimaging biomarkers of psychosis risk [2, 3, 5]. Decreased cortical thickness in the left superior temporal gyrus, right cingulate cortex, and right primary sensory and motor cortices has been associated with psychotic symptoms in the largest study of 22q11.2DS to date [6]. Widespread differences in rs-fMRI connectivity have been associated with psychosis symptoms in 22q11.2DS, encompassing frontoparietal areas, the default mode (DM) network, anterior cingulate cortex, fusiform cortex, inferior temporal cortex, and higher order visual areas [7–9]. These neuroimaging phenotypes are difficult to unify under a common mechanism for the emergence of psychosis.

In-depth cognitive phenotyping of individuals with 22q11.2DS suggests that deficits in face memory, affective processing, and social cognition stand out against a backdrop of global cognitive dysfunction [10]. In healthy adults, facial affect processing relies on the coordination of visual and emotion processing, top-down and bottom-up attention, and memory encoding and retrieval [11, 12]. These cognitive processes are subserved by temporally coordinated, evoked activity within a distributed network of limbic, insular, visual, and medial and lateral prefrontal brain areas [11, 13, 14]. Neuroimaging studies have implicated early top-down inhibition from anterior cingulate cortex to the amygdala in facial affect processing [14, 15]. Nevertheless, it remains unclear how regional activations and network interactions result in behaviorally relevant emotion processing, which hinders targeted study of dysfunctional facial affect processing in 22q11.2DS.

Multi-modal neuroimaging phenotypes in 22q11.2DS have neither provided clear explanations for the observed abnormalities in facial affect processing nor identified candidates for targeted intervention [1, 3]. Greater amygdala volumes on T1 imaging are associated with anxiety in 22q11.2DS [16]. Resting state fMRI (rs-fMRI) studies have found differences in DM network [9, 17] and frontolimbic connectivity, the latter of which correlates with anxiety [8], suggesting that frontolimbic dysconnectivity is relevant to affect processing in 22q11.2DS. Task-based fMRI studies of facial affect processing in 22q11.2DS have revealed reduced amygdalar fear accommodation and fusiform gyrus activation [18, 19]; however, these studies are limited by their focus on univariate activation measures, given that facial affect processing inherently relies on interactions among brain regions.

Here, we hypothesized that primary visual and motor processing would be preserved in individuals with 22q11.2DS, while frontolimbic interactions subserving bottom-up emotion-processing [11, 12] would be disrupted, either spatially or temporally, in individuals with 22q11.2DS. We applied constrained principal component analysis (CPCA) [20–25] to identify brain activation patterns evoked by images of faces, and quantified their time course of activation after emotion identification. Specifically, we used emotion identification task fMRI data [13, 26, 27] acquired from 58 individuals with 22q11.2DS identified through the 22q and You Center at the Children’s Hospital of Philadelphia, examined as part of a prospective brain-behavior study of 22q11.2DS, and 58 age-, sex-, and race-matched healthy controls (HCs) from the Philadelphia Neurodevelopmental Cohort (PNC) [28, 29]. The spatial profiles of task-evoked activation patterns were similar between groups, but their temporal profiles were altered in 22q11.2DS, implicating selective dysfunction in putative motor feedback (PC2) and emotional memory (PC5) signals. PC2 and PC4 activation were most strongly associated with task performance within the 22q11.2DS sample. Finally, we quantified the alignment between these task-evoked spatial activation patterns and spatial maps of gray matter structural change in individuals with 22q11.2DS. Collectively, these findings shed light on the dynamic interactions between visual, attentional, limbic, and motor systems during facial affect processing and distinguish between affected and relatively unaffected task-relevant neural systems in individuals with 22q11.2DS.

## Methods

### Participants

Emotion identification task fMRI data were obtained from a sample of 58 individuals with genotype-confirmed chromosome 22q11.2DS evaluated by the 22q and You Center at the Children’s Hospital of Philadelphia and the PNC [28], a large community-based study of brain development (see Table 1). Informed consent was obtained for all participants. Here, we study a sample of *n* = 58 age-, sex-, and race-matched PNC subjects without radiologic abnormalities or medical problems that might impact brain function. All subjects in this sample had a mean framewise displacement <0.7 mm during the emotion identification task to minimize motion-related confounds.

**Table 1:** Sample characteristics.

	22q11.2DS	PNC	*p* value
*Demographics*			
Age (y)	20.3 ± 4.8	19.6 ± 3.9	0.38
Male	50%	50%	–
White	81%	75.90%	0.58
African American	12.10%	17.20%	0.51
Other Race	6.90%	6.90%	1
CNB Accuracy (z)	−1.2	0.22	8.3 × 10^−20^
Typical or Atypical Antipsychotics, n (%)	5 (8.6%)	–	–
Mood Stabilizers, n (%)	3 (5.2%)	–	–
SNRIs/SSRIs, n (%)	11 (19%)	–	–
Stimulants, n (%)	6 (10%)	–	–
Anticholinergics, n (%)	1 (1.7%)	–	–
Benzodiazepines, n (%)	2 (3.4%)	–	–
*Imaging*			
Mean Framewise Displacement (mm)	0.119 ± 0.077	0.0762 ± 0.085	0.0057
Total Brain Volume (cm^3^)	1110 ± 120	1220 ± 120	2.3 × 10^−6^
*Task Performance (%)*			
Correct	72.9 ± 21	90.9 ± 6.2	4.4 × 10^−8^
Incorrect	17.4 ± 15	6.73 ± 4.6	3.9 × 10^−6^
NR	7.92 ± 13	2.31 ± 4.3	0.0033
Threat Correct	70.1 ± 23	89.1 ± 10	2.8 × 10^−7^
Threat Incorrect	19.6 ± 18	8.33 ± 7.6	4.7 × 10^−5^
Threat NR	8.48 ± 13	2.56 ± 5.7	0.0029
Non-Threat Correct	74.8 ± 21	92.1 ± 5	1.5 × 10^−7^
Non-Threat Incorrect	15.9 ± 15	5.66 ± 3.9	4.7 × 10^−6^
Non-Threat NR	7.54 ± 14	2.14 ± 4.1	0.007

The *p* value column was generated using two independent sample *t*-tests, except for proportions of race, which were generated by comparing bootstrapped confidence intervals of sample proportions of each race. All values, except race, sex, and medications are represented as a mean ± standard deviation. CNB, mean *z*-scored accuracy across all Penn Computerized Neurocognitive Battery sections as a surrogate for intelligence quotient [66].

*NR* no response.

### Emotion identification task

As previously described [13, 26, 27], the emotion identification task employed a fast event-related design with a jittered inter-stimulus interval (ISI). Subjects viewed 60 faces displaying neutral, happy, sad, angry, or fearful expressions, and were asked to label the emotion displayed. Stimuli construction and validation are detailed elsewhere [30]. Briefly, the stimuli were color photographs of actors (50% female) who volunteered to participate in a study on emotion. They were coached by professional directors to express a range of facial expressions. For the present task, a subset of intense expressions was selected based on high degree of accurate identification (80%) by raters. Each face was displayed for 5.5 s followed by a variable ISI of 0.5–18.5 s, during which a crosshair (matching the faces’ perceptual qualities) was displayed. Total task duration was 10.5 min.

### Structural and functional image processing

We used *fMRIprep* software [31] to perform brain extraction and segmentation of the individual high-resolution T1-weighted images, registration of task fMRI BOLD volumes to individual-specific T1 images, and computation of confound time series (see Supplementary Information for *fMRIprep* standardized methods section). After the above steps were completed using *fMRIprep* software [31], we used XCP engine [32] to perform the following steps: (1) demeaning to remove linear or quadratic trends, (2) first-order Butterworth filtering to retain signal in the 0.01–0.50 Hz range, and (3) confound regression of six realignment parameters. Following these preprocessing steps, we extracted parcellated, regional time series from the unsmoothed voxel-level data using the 200-node Schaefer cortical atlas [33] and 14 subcortical nodes defined by the Harvard-Oxford atlas [34].

### Extracting task-relevant spatiotemporal modes of brain activity through constrained principal component analysis

After completing the outlined preprocessing steps, we used constrained principal components analysis (CPCA) [24, 25] to extract task-evoked spatial modes of brain activation at the group-level with subject-level temporal weights [20–23]. Briefly, this approach involves using a finite impulse response (FIR) basis set [35] to extract task-related variance from a set of BOLD time-series, applying principal component analysis (PCA) to extract orthogonal spatiotemporal modes from the task-related variance, and then a second regression step using the same FIR basis set to determine how the temporal scores of each PC fluctuate with stimulus presentation. Here, our FIR basis set contained an indicator variable for each image acquisition spanning 0–18 s after each of six task events, consisting of correct, incorrect, and non-responses to threatening and nonthreatening stimuli. See Supplementary Information for additional details and mathematical formulation.

### Multilevel growth models of principal component response curves

In order to compare the activation of each CPCA component evoked by each task event between HCs and individuals with 22q11.2DS, we applied a multilevel growth modeling approach. This approach allowed us to account for the multilevel nature of the data, with multiple time points of component activity for different stimuli nested within participants, as well as between-subject factors such as age and sex. Briefly, for each component, we used the *nlme* package in R to fit a linear mixed effects model predicting the estimated score of that component at time *t* after each task event, excluding the two non-response task events. All models included age, sex, total brain volume, mean framewise displacement during task scans, handedness, and group membership (22q11.2DS or control). All models additionally included random intercepts to capture between-person differences in mean levels of component scores.

Next, a partially supervised model selection procedure motivated by a previous study [36] was implemented in order to include fixed effects of time, polynomials of time, stimulus type, response type, and interactions between those variables and 22q11.2DS status, when the inclusion of these variables improved model fit. We also included random effects of time to model between-person differences in how activity changed across time. See Supplementary Information, “Multilevel growth model selection procedure” for more details.

## Results

### Identifying brain activation patterns evoked by emotion identification

**Fig. 1:**
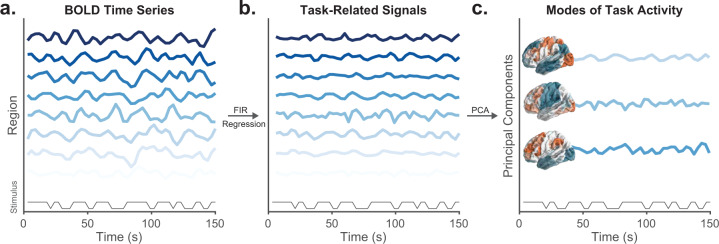
Schematic of methods for functional image analysis. **a** Example time series of BOLD signal from seven arbitrarily chosen regions acquired during an emotion identification task. Boxcar regressor of stimulus presentation is shown below the BOLD signal. **b** In order to isolate task-related signals, the BOLD signal from (**a**) is regressed onto a finite impulse response basis set, which flexibly captures each region’s response to different stimuli without assuming any particular shape of the hemodynamic response function. **c** The predicted values of the linear regression model are decomposed with principal component analysis, yielding orthogonal spatial maps of task-evoked brain activity with orthogonal temporal profiles. These spatiotemporal modes can be related back to stimulus presentation in order to estimate the task evoked time course of each spatial activation pattern. *FIR* finite impulse response. *PCA* principal component analysis.

**Fig. 2:**
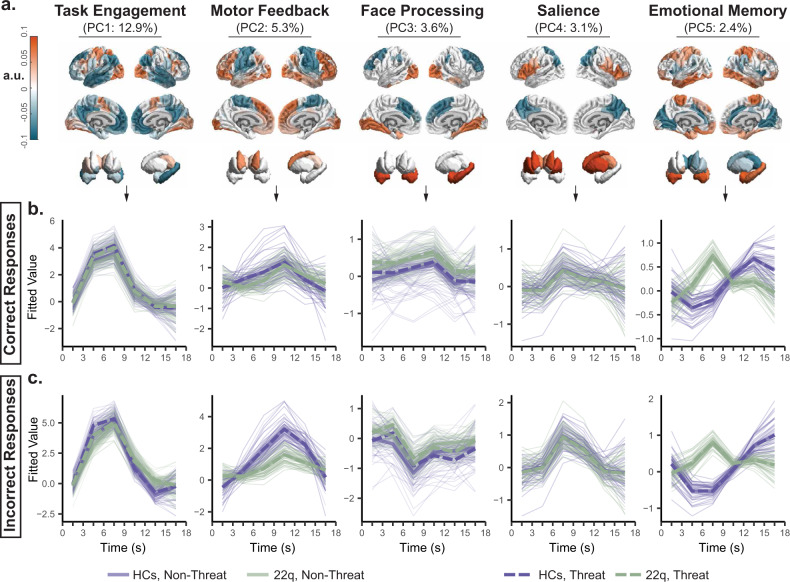
Spatiotemporal modes of activity evoked by emotion identification are selectively altered in 22q11.2DS. **a** Spatial loadings of the first five principal components of task-related variance (Fig. 1b) in emotion identification task BOLD signal thresholded at *p* < 10^−4^ using bootstrap significance testing [65], shown on surface renderings of cortex and subcortex. Components are named based upon the authors’ interpretation of the data and existing literature on localization of brain function (see Discussion). **b**, **c** Multilevel growth models fit to the temporal scores (*y*-axis) of each task-evoked PC during the time (*x*-axis) occurring 0–18 s after correct (**b**) or incorrect (**c**) emotion identification of threatening (thick lines) and nonthreatening (dashed lines) faces. We used a model selection procedure (see Methods) to predict each PC’s scores over time from polynomials of time, stimulus type (threat or non-threat), response type (correct or incorrect), 22q status, and interactions between those variables while controlling for age, sex, total brain volume, head motion, and handedness. The best model selected through this process was used to obtain fitted values (*y*-axis) to describe the trajectory of each PC’s score for the prototypical individual in each group (thick, opaque lines) and for each participant (thin, faded lines).

Individuals with 22q11.2DS exhibit deficits in facial affect processing and social cognitive function. However, the dynamic patterns of brain activation underlying these deficits are not fully understood. Here, we conducted a spatiotemporally sensitive analysis of task-related brain activity using CPCA [24] to analyze BOLD data from 58 individuals with 22q11.2DS and 58 age-, sex-, and race-matched HCs. First, we regressed BOLD signal (Fig. 1a) from an emotion identification task onto a FIR basis set to extract stimulus-related signals (Fig. 1b). We used separate regressors for each subject and four *task events* of interest, in which threatening or nonthreatening stimuli were accompanied by either correct or incorrect responses [37, 38]. Next, to complete the CPCA procedure, we identified the principal components of the task related variance in BOLD signal captured by the predicted values of this regression model (Fig. 1c). A scree plot of the variance explained by this PCA revealed an elbow at six components, which cumulatively explained 64.1% of the task-related variance in the BOLD signal (Fig. S1a). The first principal component (Fig. S2), explaining 36.7% of task-related variance, appeared to reflect a global signal fluctuation [39], and was thus excluded from further analysis. We named this global signal component “PC0” and re-indexed the original PC2-6 as PC1-5 for future analyses. Finally, we applied a bootstrapping analysis (see Supplementary Information, subsection “Bootstrapping analysis of CPCA components”) to threshold these spatial maps (Fig. 2a) and demonstrate that a group CPCA solution was adequate to describe each cohort’s BOLD data (Fig. S3a), suggesting that spatial differences in activation between the groups are relatively small. Collectively, these analyses revealed multiple task-evoked spatial activity patterns that occur in both HCs and individuals with 22q11.2DS.

### Altered temporal profiles of task-evoked brain activity in 22q11.2DS

After identifying spatial patterns of task-related brain activity, we next sought to characterize each signal component’s evoked response to the four *task events*. We regressed PC scores onto an FIR basis set to estimate the mean score of each PC at the six image acquisitions occurring 0–18 s after each task event (Fig. S4a, c). Next, we applied a model selection procedure using multilevel growth models to parameterize the shape of each PC’s event response curve with polynomial functions of time (Fig. 2b, c; see Methods). This analysis allowed us to statistically compare the temporal profiles of these PC response curves between HCs and 22q11.2DS individuals while accounting for effects and interactions (Supplementary Data File 1) of between-subject factors (total brain volume, sex, age, head motion, and handedness) and within-subject factors (task event). Notably, results were robust to parcellation scheme (Fig. S5) and no activation was detected when BOLD data were phase randomized to create stimulus-independent surrogate null data (Fig. S6). In addition, our results were similar when we only studied 64 subjects with overall accuracy ≥75% (Fig. S7).

First, we observed that PC1 was rapidly and robustly engaged in each task event, peaking around 7.5 s after task event onset (Fig. 2b, c, leftmost subpanel). The spatial map of PC1 revealed DM network deactivation [40, 41], visual cortex activation, and left-hemispheric hand motor cortex activation. The temporal expression of PC1 was highest during correct responses to threat stimuli (Fig. 2b, c; Time^3^ × Threat × Correct, *β* = 4.8 × 10^−3^, *p* = 3.7 × 10^−3^, df = 2200), but primarily differed between HCs and 22q11.2DS during incorrect responses and less so during correct responses (Fig. 2b, c; Time^2^ × 22q × Correct, *β* = 0.021, *p* = 1.5 × 10^−3^, df = 2200). These findings support our hypothesis that activation of unimodal visual and somatomotor cortex would be relatively preserved in individuals with 22q11.2DS.

Next, we observed that PC2 showed the most pronounced activation during incorrect responses (Fig. 2b, c; Time^2^ × Correct, *β* = 0.037, *p* = 5.2 × 10^−16^, df = 2200). The PC2 peak was delayed, occurring around 10.5 s after the task event in contrast to the peak at 7.5 s observed in PC1. The spatial map of PC2 consisted of dorsolateral and ventrolateral prefrontal cortex activation amid low amplitude activity in sensorimotor areas. Notably, we found an interaction between 22q11.2DS status, time, and response type such that 22q11.2DS showed reduced activation of PC2 during incorrect responses (Fig. 2b, c; Time^2^ × 22q × Correct, *β* = −0.031, *p* = 6.3 × 10^–7^, df = 2200). This finding was unexpected given our hypothesis that early-activating regions would be affected in individuals with 22q11.2DS.

PC3 activity showed a positive peak around 10.5 s during correct responses and a negative peak at 7.5 s during incorrect responses, with the greatest responses to threatening stimuli (Fig. 2b, c; Time^2^ × Correct × Threat, *β* = −0.012, *p* = 0.031, df = 2200). The spatial map of PC3 showed activation of the amygdala, hippocampus, and fusiform gyrus, with activity decreases in dorsolateral prefrontal regions (Fig. 2a). In 22q11.2DS, activation of this component was higher at baseline (Fig. 2b, c; 22q, *β* = 0.29, *p* = 1.2 × 10^−3^, df = 96), apparently capturing the attenuated decrease of this component during incorrect response (Fig. S4c, third panel from right).

PC4 peaked early around 7.5 s after the task event. The spatial map of PC4 was characterized by activation in the bilateral opercula, insulae, and motor basal ganglia with low amplitude activity in the posterior cingulate and posterior parietal cortex (Fig. 2a). Stimulus type was not associated with PC4’s time course, but the response was more pronounced during incorrect trials. (Fig. 2b, c; Time^3^ × Correct, *β* = −2.2 × 10^−3^, *p* = 5 × 10^−4^, df = 2200). There was a trend toward reduced PC4 expression during correct non-threat trials in HCs only (Fig. S4a, 4th panel from the left), but models containing time-by-stimulus-by-response-by-cohort interaction coefficients did not meet statistical significance. Overall, we did not detect any statistically significant group differences in the temporal response of PC4.

Finally, PC5 exhibited a biphasic activation profile, with an early negative peak around 4.5 s and a delayed positive peak around 13.5 s after the task event in HCs (Fig. 2b, c; Time^5^, *β* = 8 × 10^−5^, *p* = 0.012, df = 2200). However, individuals with 22q11.2DS had only one early peak around 7.5 s (Fig. 2b, c; Time^4^ × 22q, *β* = −5.5 × 10^−4^, *p* = 5.4 × 10^−4^, df = 2200). The spatial map of PC5 showed engagement of the hippocampus, amygdala, entorhinal cortex, ventromedial prefrontal cortex, and bilateral hand motor sensorimotor cortices with suppression of thalamus, anterior cingulate cortex, and insula (Fig. 2a). These findings supported our hypothesis that early, frontolimbic interactions would be disrupted in individuals with 22q11.2DS.

### Individual differences in activation peaks explain variance in task performance within 22q11.2DS sample

Next, we were interested to understand the relevance of these spatiotemporal modes of brain activation to cognitive function within the 22q11.2DS population. We used each 22q11.2DS individual’s peak score on each of the five components during each of the four task events as independent variables in separate models to predict the rank of in-scanner accuracy on the emotion identification task under study. We used the rank of accuracy as our outcome variable rather than the percentage accuracy in order to include as many 22q11.2DS individuals as possible without biasing regression estimation with outlier values. Each of these 20 models included age, sex, total brain volume, mean task-scan head motion, and handedness as covariates.

**Fig. 3:**
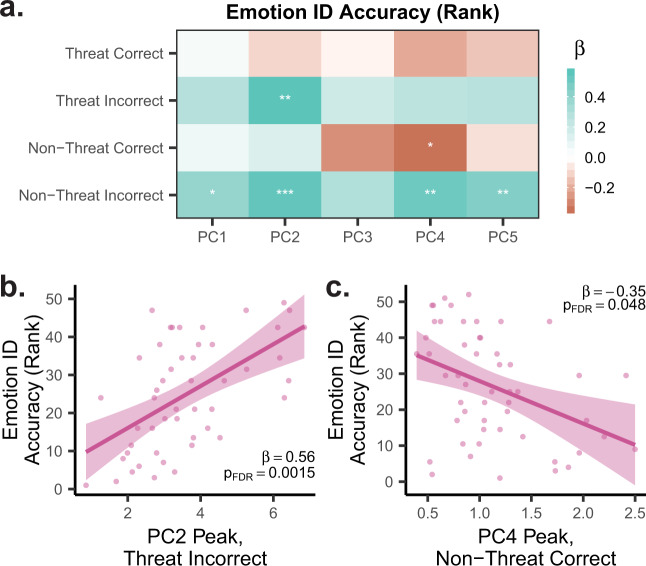
Overall task performance in individuals with 22q11.2DS can be predicted from peak PC scores. **a** Standardized linear regression *β* weights (color axis) for the peak value of each PC (*x*-axis) during each task event (*y*-axis) as a predictor of overall in-scanner emotion identification accuracy using the sample of individuals with 22q11.2DS only, in a model containing age, sex, total brain volume, head motion, and handedness as covariates. Asterisks indicate level of significance after FDR correction (*q* < 0.05) overall 20 *β* values: *, *p*_FDR_ < 0.05. **, *p*_FDR_ < 0.01. ***, *p*_FDR_ < 0.001. **b**, **c** Partial residuals of emotion identification accuracy (*y*-axis) from linear regression models in (**a**) plotted against peak PC2 scores during incorrect responses to nonthreatening stimuli (**b**) or peak PC4 scores during correct responses to nonthreatening stimuli (**c**) (*x*-axis).

This analysis revealed that PC2 and PC4 scores were the most strongly associated with correct emotion identification in 22q11.2DS individuals. Specifically, we found that PC2 peak values during threat incorrect (Fig. 3a; *β* = 0.55, *p*_FDR_ = 0.0022, df = 42) and non-threat incorrect trials (Fig. 3a, b; *β* = 0.63, *p*_FDR_ = 1.99 × 10^−5^, *df* = 43) were positively associated with emotion identification accuracy. PC4 peak values during non-threat correct trials were negatively associated with accuracy (Fig. 3a, c; *β* = −0.45, *p*_FDR_ = 0.0075, df = 45), whereas PC4 peak values during non-threat incorrect trials were positively associated with accuracy (Fig. 3a; *β* = 0.49, *p*_FDR_ = 0.0055, df = 43). These associations were weaker but still present in controls (Fig. S8a, c), and no interaction term between PC peak and group was statistically significant. These findings suggest that the presence of opposing frontal-motor activation (Fig. 2, PC2) during incorrect trials and insular activation (Fig. 2, PC4) during incorrect trials but not correct trials index accurate emotion identification in 22q11.2DS.

### Differences in brain structure in 22q11.2DS selectively align with task-evoked activation patterns

After characterizing functional brain abnormalities during emotion identification in 22q11.2DS, we examined whether differences in gray matter morphometry could be a substrate for these functional effects. Here, we tested the hypothesis that areas with abnormal cortical morphometry in 22q11.2DS align with the identified task-evoked activation patterns, possibly hindering the function of regions that are specifically engaged during emotion identification (Fig. 2b–e).

**Fig. 4:**
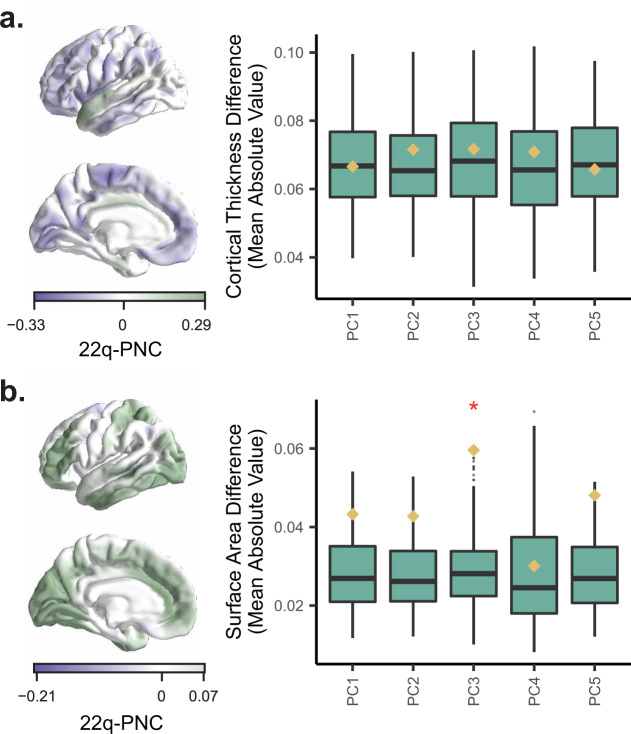
Differences in cortical surface area in 22q11.2DS align with face processing component. **a**–**b** Surface plots show cortical thickness (**a**) and cortical surface area (**b**) differences between HCs and individuals with 22q11.2DS, reproduced with permission from [6]. Yellow diamonds show mean absolute value (MAV) of cortical thickness (**a**) and cortical surface area (**b**) differences within the areas of each spatial PC map (Fig. 2a) that differed from 0 after bootstrap thresholding at *p* < 10^−4^. The green boxplots show the same measure of MAV within each PC map computed using 500 permuted versions of the structural maps with preserved spatial covariance [42]. Red *, *p*spin_FDR_ < 0.05, corrected over 12 comparisons for six PCs and two structural maps.

To test this hypothesis, we utilized difference maps of cortical thickness (Fig. 4a) and cortical surface area (Fig. 4b) obtained from a previously published manuscript [6] using a larger, partially overlapping sample (42 of the 22q11.2DS subjects and 11 of the PNC control subjects studied here). Importantly, cortical thickness and surface area can only be computed from the cerebral cortex, and thus subcortical structures are excluded from this analysis. We computed the mean absolute value (MAV) of structural change for each metric within the cortical areas of each spatial PC map for which the loading value was significantly different from 0 after bootstrap thresholding (Fig. 2a, *p* < 10^−4^. MAV captures the total extent of structural differences, encompassing both increases and decreases in cortical thickness or surface area, within activated or deactivated regions for each PC. We compared the MAV values (Fig. 4a, b, yellow diamonds) to a null distribution of MAV values obtained using 500 permuted structural maps with preserved spatial covariance [42]. This analysis revealed that PC3 harbored differences in cortical surface area within its engaged areas that were greater than expected due to spatial covariance alone (Fig. 4b; MAV = 0.059, *p*_spinFDR_ < 0.002). The MAV of cortical thickness within any PC map did not differ from that which would be expected due to spatial covariance (Fig. 4a; all *p*_spinFDR_ > 0.05). These findings suggest that differences in cortical surface area, rather than cortical thickness, align more specifically with activation patterns associated with face processing.

## Discussion

In the present study, we extracted five spatial patterns of task-evoked brain activity from individuals with 22q11.2DS and matched HCs. These activation patterns appeared to engage both “task-general” (PC1, PC2, and PC4) systems that are seen across many tasks, as well as “emotion-related” (PC3 and PC5) systems, which are more specifically engaged during facial affect processing tasks. We found the strongest group differences in PC2 and PC5. Finally, we showed cortical gray matter surface area differences in 22q11.2DS aligned with the spatial map of PC3, due to engagement of primary visual cortex, inferotemporal cortex, and dorsolateral prefrontal cortex.

### Altered task-general brain dynamics in 22q11.2DS

Of the three task-general components, we found that PC1 and PC4 were relatively preserved in 22q11.2DS. PC1 contained rapid engagement of dominant hand motor cortex with visual cortex activation and DM deactivation observed across all task events. Default mode (DM) deactivation is a hallmark of goal oriented tasks [41]. Prior fMRI studies in 22q11.2DS have found both decreased and increased spontaneous activity in DM subregions [7, 9]. In the task fMRI setting studied here, we find timing dependent differences. The DM is relatively unaffected in the early response (PC1) with more group differences in DM subregions in the delayed response (PC2 and PC5). PC4 was characterized by early-peaking insular activation, most robust after incorrect responses to potentially unfamiliar or ambiguous stimuli, consistent with the insula’s role in detecting novel stimuli [43]. Emotion identification accuracy was negatively associated with PC4 activation during incorrect trials within the 22q11.2DS sample, suggesting that inappropriate, early insular responses to stimuli may contribute to or reflect poor task performance.

The remaining task-general component implicates aberrant motor feedback in 22q11.2DS during failures of emotion identification. In HCs, PC2 was more strongly engaged during incorrect than correct responses and consisted of delayed frontal activation with sensorimotor deactivation. We interpreted these findings as a negative feedback signal from bilateral inferior frontal gyri to motor cortex. This pattern is consistent with the known role of the inferior frontal gyrus in response inhibition [44]. The lack of this signal was associated with poor emotion identification accuracy in the 22q11.2DS sample, consistent with previously observed motor dysfunction in individuals with 22q11.2DS [45–47]. This relationship was weaker but present in controls, suggesting the 22q11.2DS inviduals lie on the lower end of a spectrum of pathologic activation of PC2. However, given that subjects are not notified of incorrect responses during this task, PC2 activation may also be explained by a lack of post-response recognition of an incorrect choice in 22q11.2DS individuals.

### Altered emotion-related brain dynamics in 22q11.2DS

Individuals with 22q11.2DS show deficits in social cognition and face memory even after adjusting for global cognitive deficits [10]. fMRI studies of face processing in 22q11.2DS have found hypoactivation of fusiform gyrus and a lack of amygdalar fear accommodation [18]. Here, we found altered time courses of PC3 and PC5, which both engaged fusiform gyrus, amygdala, and hippocampus. PC3 also revealed dorsolateral prefrontal cortex deactivation and peaked at 10.5 s during correct responses. Interestingly, a negative PC3 peak occurred 7.5 s after incorrect responses, implicating suppression of face processing circuitry and activation of dorsal attention areas in incorrect responses, an effect that was less pronounced in 22q11.2DS. This finding may reflect incorrect responses in HCs resulting from futile goal-directed cognition amidst failure of limbic processing, while individuals with 22q11.2DS may experience failure of limbic processing with less compensatory goal-directed cognition. In addition, the spatial map of PC3 showed statistically significant alignment with cortical surface area alterations in 22q11.2DS, which may explain the abnormal temporal profile of PC3; however, the observed differences were small, and therefore structural alterations may instead alter local processing despite relatively normal onset and activation magnitude. Abnormal local processing could in turn affect the engagement of concurrently (PC2) or later peaking (PC5) components.

In addition to the primary sensory processing underlying facial recognition, emotional memory [48] contributes to facial affect processing and engages a similar set of brain areas [12]. PC5 harbored thalamic deactivation and a delayed peak around 13.5 s, suggesting that this component may reflect memory encoding rather than retrieval in HCs; however, in individuals with 22q11.2DS, this component peaked early at 7.5 s with an absent late peak. This early peak may reflect inappropriate early activation of emotional circuitry and the absence of a late peak may reflect dysfunctional emotional memory encoding. Indeed, emotional memory deficits in a mouse model of 22q11.2DS have been linked to disrupted thalamo-amygdalar signaling [49]. Collectively, PC3 and PC5 may provide separable measures of dysfunction in affective processing in individuals with 22q11.2DS.

### Methodological limitations

Though this study provides a great deal of information about spatiotemporal patterns of task-evoked brain activity in 22q11.2DS, several key limitations must be acknowledged. First, the fact that global cognitive deficits are observed in individuals with 22q11.2DS raises the possibility that reduced task engagement may confound our observations of abnormal task-related brain activity. While we cannot eliminate this possibility, the relative similarity in PC1 activation between groups suggests that primary visual processing, DM deactivation, and motor execution are intact in 22q11.2DS. Second, PCA enforces a spatiotemporally orthogonal solution, a constraint that is not biologically necessitated. Future studies could explore this limitation by benchmarking PCA solutions against varimax-rotated PCA, non-negative matrix factorization, or other non-orthogonal decompositions. Finally, individuals with 22q11.2DS exhibit increased in-scanner head motion. Our motion exclusion threshold (mean framewise displacement <0.7 mm), which was lenient relative to the threshold of 0.2 mm [50] recommended for HCs, may have biased our sample toward less severe phenotypes in 22q11.2DS. This threshold is more stringent compared to previous studies of facial affect processing task fMRI in 22q11.2DS [18, 19], and it is difficult to compare to rs-fMRI studies of 22q11.2DS [7–9, 17]. We attempted to address any remaining motion contamination by including mean framewise displacement as a covariate in subsequent regression analysis.

### Future directions

In the future, targeted task design would enhance the interpretation of these signals in relation to emotional cognition in 22q11.2DS. For instance, one could follow the emotion identification task with a face recognition task [37]. If PC5 scores during emotion identification predicts future correct recognition, one could infer that PC5 reflects memory encoding. This task would also allow separation of components involved in emotion identification from those involved in emotion perception. To investigate the relationship between PC2 and motor feedback, one could test whether notification of errors modifies the response of PC2 during incorrect trials.

In the present study, our comparison of structure and function was limited to gray matter differences, though it has been shown that the dynamic spreading of activation along white matter tracts supports task-related and spontaneous fluctuations in brain activity [51–53]. Network control theory [54–56] provides tools that account for both external inputs, such as task stimuli, and internal spreading dynamics along white matter connections. One recent study found that control properties of structural brain networks explained dysfunctional resting state connectivity in 22q11.2DS [57]; future studies could apply these tools to assess the temporal alterations in stimulus-driven brain activity identified here.

## Citation diversity statement

Recent work in several fields of science has identified a bias in citation practices such that papers from women and other minorities are under-cited relative to the number of such papers in the field [58–63]. Here we sought to proactively consider choosing references that reflect the diversity of the field in thought, form of contribution, gender, and other factors. We obtained predicted gender of the first and last author of each reference by using databases that store the probability of a name being carried by a woman [58, 64]. By this measure (and excluding self-citations to the first and last authors of our current paper), our references contain 12.1% woman(first)/woman(last), 7.6% man/woman, 21.2% woman/man, and 59.1% man/man. This method is limited in that (a) names, pronouns, and social media profiles used to construct the databases may not, in every case, be indicative of gender identity and (b) it cannot account for intersex, non-binary, or transgender people. We look forward to future work that could help us to better understand how to support equitable practices in science.

## Supplementary information

Supplementary Data File 1

Supplement

## Data Availability

Structural and functional neuroimaging data for PNC subjects are available at https://www.ncbi.nlm.nih.gov/projects/gap/cgi-bin/study.cgi?study_id=phs000607.v3.p2.

## Code availability

All analysis code is available at https://github.com/ejcorn/fir_pca_22q.
