# Supplementary material for: Altered functional brain dynamics in chromosome 22q11.2 deletion syndrome during facial affect processing
Source: Mol Psychiatry. 2021 Oct 22;27(2):1158–66. doi: 10.1038/s41380-021-01302-y (PMC9023602; doi:10.1038/s41380-021-01302-y)
Supplement: Supplementary file 1 — Supplementary Data File 1 [file 41380_2021_1302_MOESM1_ESM.docx]

**Supplementary Data File 1: Mixed Effects Model Coefficient Tables**

| **PC0** |  | Fixed Effects | |  |  |
| --- | --- | --- | --- | --- | --- |
| Coefficient Name | Estimate | Std.Error | DF | t-value | p-value |
| (Intercept) | 5.4 | 3.5 | 1387 | 1.6 | 0.12 |
| Age (months) | 0.00088 | 0.0034 | 57 | 0.26 | 0.8 |
| Sex (Female) | 0.21 | 0.31 | 57 | 0.7 | 0.49 |
| Total Brain Volume | 7.20E-07 | 1.50E-06 | 57 | 0.49 | 0.63 |
| Mean Head Motion | -0.067 | 1.7 | 57 | -0.04 | 0.97 |
| Handedness | -0.39 | 0.33 | 57 | -1.2 | 0.24 |
| 22q Status | 0.17 | 0.38 | 57 | 0.45 | 0.66 |
| Time | -9.3 | 2.7 | 1387 | -3.5 | 0.00056 |
| Time^2 | 3.6 | 0.87 | 1387 | 4.2 | 3.00E-05 |
| Time^3 | -0.52 | 0.12 | 1387 | -4.4 | 1.10E-05 |
| Time^4 | 0.032 | 0.0072 | 1387 | 4.4 | 1.20E-05 |
| Time^5 | -0.00068 | 0.00016 | 1387 | -4.3 | 2.10E-05 |
|  |  |  |  |  |  |
|  |  |  |  |  |  |
|  |  | Random Effects | |  |  |
| Coefficient Name | lower | est. | upper |  |  |
| Intercept | 0.74151966 | 1.39682773 | 2.63125554 |  |  |
| Time | 0.40672317 | 0.59317854 | 0.86511121 |  |  |
| Time^2 | 0.03128514 | 0.04236985 | 0.057382 |  |  |

| **PC1** |  | Fixed Effects | |  |  |
| --- | --- | --- | --- | --- | --- |
| Coefficient Name | Estimate | Std.Error | DF | t-value | p-value |
| (Intercept) | 3 | 1.9 | 1365 | 1.6 | 0.11 |
| Age (months) | -2.00E-04 | 0.0015 | 57 | -0.13 | 0.9 |
| Sex (Female) | 0.012 | 0.14 | 57 | 0.085 | 0.93 |
| Total Brain Volume | 5.50E-07 | 6.50E-07 | 57 | 0.85 | 0.4 |
| Mean Head Motion | -0.62 | 0.76 | 57 | -0.82 | 0.42 |
| Handedness | 0.084 | 0.15 | 57 | 0.56 | 0.58 |
| 22q Status | 0.99 | 0.87 | 57 | 1.1 | 0.26 |
| Time | -5.1 | 1.5 | 1365 | -3.5 | 0.00051 |
| Time^2 | 2.4 | 0.44 | 1365 | 5.4 | 6.70E-08 |
| Time^3 | -0.37 | 0.058 | 1365 | -6.3 | 4.20E-10 |
| Time^4 | 0.022 | 0.0035 | 1365 | 6.4 | 1.90E-10 |
| Time^5 | -0.00047 | 7.70E-05 | 1365 | -6.2 | 8.80E-10 |
| Correct | -0.74 | 1.5 | 1365 | -0.51 | 0.61 |
| Threat | -3.6 | 1.4 | 1365 | -2.6 | 0.0087 |
| Time:Correct | 0.72 | 1.1 | 1365 | 0.66 | 0.51 |
| Time:Threat | 3.4 | 1.1 | 1365 | 3.1 | 0.002 |
| Correct:Threat | 3.3 | 1.9 | 1365 | 1.7 | 0.082 |
| Time^2:Correct | -0.32 | 0.24 | 1365 | -1.3 | 0.18 |
| Time^2:Threat | -0.76 | 0.24 | 1365 | -3.1 | 0.0018 |
| Time^3:Correct | 0.037 | 0.02 | 1365 | 1.9 | 0.059 |
| Time^3:Threat | 0.061 | 0.02 | 1365 | 3 | 0.0023 |
| Time^4:Correct | -0.0012 | 0.00054 | 1365 | -2.3 | 0.024 |
| Time^4:Threat | -0.0016 | 0.00056 | 1365 | -2.9 | 0.0033 |
| Is22q:Time | -0.98 | 0.41 | 1365 | -2.4 | 0.016 |
| Is22q:Time^2 | 0.16 | 0.052 | 1365 | 3.1 | 0.002 |
| Is22q:Time^3 | -0.0062 | 0.0019 | 1365 | -3.3 | 0.001 |
| Is22q:Correct | -0.86 | 1.1 | 1365 | -0.78 | 0.44 |
| Time:Correct:Threat | -3.2 | 1.5 | 1365 | -2.1 | 0.035 |
| Time^2:Correct:Threat | 0.77 | 0.33 | 1365 | 2.3 | 0.022 |
| Time^3:Correct:Threat | -0.066 | 0.028 | 1365 | -2.4 | 0.018 |
| Time^4:Correct:Threat | 0.0018 | 0.00077 | 1365 | 2.4 | 0.018 |
| Is22q:Time:Correct | 0.94 | 0.55 | 1365 | 1.7 | 0.087 |
| Is22q:Time^2:Correct | -0.16 | 0.071 | 1365 | -2.2 | 0.029 |
| Is22q:Time^3:Correct | 0.0061 | 0.0026 | 1365 | 2.3 | 0.019 |
|  |  |  |  |  |  |
|  |  |  |  |  |  |
|  |  | Random Effects | |  |  |
| Coefficient Name | lower | est. | upper |  |  |
| Intercept | 0.83475879 | 1.18815655 | 1.69116638 |  |  |
| Time | 0.17939219 | 0.25876505 | 0.37325678 |  |  |
| Time^2 | 0.01095438 | 0.01541823 | 0.02170107 |  |  |

| **PC2** |  | Fixed Effects | |  |  |
| --- | --- | --- | --- | --- | --- |
| Coefficient Name | Estimate | Std.Error | DF | t-value | p-value |
| (Intercept) | -2.2 | 1.6 | 1378 | -1.4 | 0.15 |
| Age (months) | -0.00067 | 0.0016 | 57 | -0.43 | 0.67 |
| Sex (Female) | -0.083 | 0.14 | 57 | -0.6 | 0.55 |
| Total Brain Volume | -6.60E-08 | 6.70E-07 | 57 | -0.099 | 0.92 |
| Mean Head Motion | -0.94 | 0.78 | 57 | -1.2 | 0.23 |
| Handedness | 0.15 | 0.15 | 57 | 1 | 0.32 |
| 22q Status | 2.6 | 0.59 | 57 | 4.4 | 5.00E-05 |
| Time | 1.8 | 1.1 | 1378 | 1.6 | 0.11 |
| Time^2 | -0.55 | 0.36 | 1378 | -1.6 | 0.12 |
| Time^3 | 0.095 | 0.049 | 1378 | 2 | 0.051 |
| Time^4 | -0.0068 | 0.003 | 1378 | -2.3 | 0.022 |
| Time^5 | 0.00016 | 6.60E-05 | 1378 | 2.5 | 0.013 |
| Correct | 1.4 | 0.58 | 1378 | 2.3 | 0.02 |
| Time:Correct | -0.21 | 0.25 | 1378 | -0.85 | 0.39 |
| Time^2:Correct | -0.044 | 0.031 | 1378 | -1.4 | 0.15 |
| Time^3:Correct | 0.0031 | 0.0011 | 1378 | 2.8 | 0.006 |
| Is22q:Time | -0.89 | 0.16 | 1378 | -5.4 | 6.60E-08 |
| Is22q:Time^2 | 0.045 | 0.0088 | 1378 | 5.2 | 2.80E-07 |
| Is22q:Correct | -2.2 | 0.63 | 1378 | -3.4 | 0.00063 |
| Is22q:Time:Correct | 0.71 | 0.16 | 1378 | 4.3 | 2.00E-05 |
| Is22q:Time^2:Correct | -0.033 | 0.0089 | 1378 | -3.7 | 0.00019 |
|  |  |  |  |  |  |
|  |  |  |  |  |  |
|  |  | Random Effects | |  |  |
| Coefficient Name | lower | est. | upper |  |  |
| Intercept | 0.99631849 | 1.37052935 | 1.88529141 |  |  |
| Time | 0.33253144 | 0.4354607 | 0.57024992 |  |  |
| Time^2 | 0.01782814 | 0.0233728 | 0.03064188 |  |  |

| **PC3** |  | Fixed Effects | |  |  |
| --- | --- | --- | --- | --- | --- |
| Coefficient Name | Estimate | Std.Error | DF | t-value | p-value |
| (Intercept) | -5.4 | 1.5 | 1381 | -3.5 | 0.00047 |
| Age (months) | 0.00032 | 0.0012 | 57 | 0.26 | 0.8 |
| Sex (Female) | -0.0045 | 0.11 | 57 | -0.041 | 0.97 |
| Total Brain Volume | 2.40E-07 | 5.30E-07 | 57 | 0.45 | 0.65 |
| Mean Head Motion | -0.029 | 0.62 | 57 | -0.047 | 0.96 |
| Handedness | 0.1 | 0.12 | 57 | 0.86 | 0.4 |
| 22q Status | 0.34 | 0.14 | 57 | 2.5 | 0.017 |
| Time | 5.6 | 1.4 | 1381 | 4.1 | 3.60E-05 |
| Time^2 | -1.9 | 0.44 | 1381 | -4.3 | 2.20E-05 |
| Time^3 | 0.25 | 0.06 | 1381 | 4.2 | 2.70E-05 |
| Time^4 | -0.015 | 0.0036 | 1381 | -4.1 | 4.20E-05 |
| Time^5 | 0.00032 | 8.00E-05 | 1381 | 4 | 6.70E-05 |
| Correct | 4.9 | 1.8 | 1381 | 2.8 | 0.0049 |
| Time:Correct | -5.4 | 1.9 | 1381 | -2.9 | 0.0038 |
| Time^2:Correct | 1.7 | 0.6 | 1381 | 2.8 | 0.0046 |
| Time^3:Correct | -0.22 | 0.082 | 1381 | -2.6 | 0.0085 |
| Time^4:Correct | 0.012 | 0.005 | 1381 | 2.4 | 0.016 |
| Time^5:Correct | -0.00025 | 0.00011 | 1381 | -2.2 | 0.026 |
|  |  |  |  |  |  |
|  |  |  |  |  |  |
|  |  | Random Effects | |  |  |
| Coefficient Name | lower | est. | upper |  |  |
| Intercept | 0.36726004 | 0.64824991 | 1.14422453 |  |  |
| Time | 0.13124755 | 0.20503708 | 0.32031228 |  |  |
| Time^2 | 0.00707138 | 0.01105588 | 0.01728552 |  |  |

| **PC4** |  | Fixed Effects | |  |  |
| --- | --- | --- | --- | --- | --- |
| Coefficient Name | Estimate | Std.Error | DF | t-value | p-value |
| (Intercept) | 2.9 | 1.1 | 1379 | 2.6 | 0.01 |
| Age (months) | 0.00012 | 0.001 | 57 | 0.12 | 0.9 |
| Sex (Female) | 0.12 | 0.09 | 57 | 1.3 | 0.2 |
| Total Brain Volume | 4.30E-07 | 4.30E-07 | 57 | 0.99 | 0.33 |
| Mean Head Motion | -0.49 | 0.5 | 57 | -0.97 | 0.34 |
| Handedness | -0.2 | 0.099 | 57 | -2 | 0.052 |
| 22q Status | -0.19 | 0.41 | 57 | -0.46 | 0.65 |
| Time | -3.8 | 0.87 | 1379 | -4.4 | 1.20E-05 |
| Time^2 | 1.1 | 0.27 | 1379 | 4.2 | 3.50E-05 |
| Time^3 | -0.14 | 0.037 | 1379 | -3.7 | 0.00026 |
| Time^4 | 0.0071 | 0.0022 | 1379 | 3.2 | 0.0016 |
| Time^5 | -0.00014 | 4.90E-05 | 1379 | -2.7 | 0.0063 |
| Correct | -0.82 | 0.61 | 1379 | -1.3 | 0.18 |
| Time:Correct | 0.86 | 0.48 | 1379 | 1.8 | 0.074 |
| Time^2:Correct | -0.24 | 0.11 | 1379 | -2.2 | 0.026 |
| Time^3:Correct | 0.022 | 0.0089 | 1379 | 2.5 | 0.012 |
| Time^4:Correct | -0.00066 | 0.00025 | 1379 | -2.7 | 0.008 |
| Is22q:Time | 0.39 | 0.18 | 1379 | 2.2 | 0.03 |
| Is22q:Time^2 | -0.054 | 0.023 | 1379 | -2.4 | 0.017 |
| Is22q:Time^3 | 0.0019 | 0.00084 | 1379 | 2.2 | 0.026 |
|  |  |  |  |  |  |
|  |  |  |  |  |  |
|  |  | Random Effects | |  |  |
| Coefficient Name | lower | est. | upper |  |  |
| Intercept | 0.543436 | 0.7329011 | 0.98842185 |  |  |
| Time | 0.06281379 | 0.08161004 | 0.10603082 |  |  |

| **PC5** |  | Fixed Effects | |  |  |
| --- | --- | --- | --- | --- | --- |
| Coefficient Name | Estimate | Std.Error | DF | t-value | p-value |
| (Intercept) | -1.7 | 0.66 | 1378 | -2.6 | 0.009 |
| Age (months) | 0.00098 | 9.00E-04 | 57 | 1.1 | 0.28 |
| Sex (Female) | -0.038 | 0.081 | 57 | -0.47 | 0.64 |
| Total Brain Volume | 4.80E-07 | 3.90E-07 | 57 | 1.2 | 0.22 |
| Mean Head Motion | 0.23 | 0.45 | 57 | 0.52 | 0.6 |
| Handedness | -0.012 | 0.089 | 57 | -0.13 | 0.9 |
| 22q Status | 1 | 0.31 | 57 | 3.3 | 0.0018 |
| Time | 0.15 | 0.022 | 1378 | 7 | 3.90E-12 |
| Correct | 0.72 | 0.28 | 1378 | 2.5 | 0.011 |
| Threat | 0.39 | 0.3 | 1378 | 1.3 | 0.2 |
| Time:Correct | -0.11 | 0.027 | 1378 | -4 | 7.60E-05 |
| Time:Threat | -0.11 | 0.029 | 1378 | -3.8 | 0.00015 |
| Correct:Threat | -0.69 | 0.4 | 1378 | -1.7 | 0.086 |
| Is22q:Time | -0.13 | 0.03 | 1378 | -4.3 | 2.10E-05 |
| Is22q:Correct | -0.64 | 0.39 | 1378 | -1.7 | 0.096 |
| Is22q:Threat | -0.46 | 0.4 | 1378 | -1.1 | 0.26 |
| Time:Correct:Threat | 0.14 | 0.039 | 1378 | 3.5 | 0.00044 |
| Is22q:Time:Correct | 0.087 | 0.037 | 1378 | 2.3 | 0.02 |
| Is22q:Time:Threat | 0.11 | 0.039 | 1378 | 2.9 | 0.0042 |
| Is22q:Correct:Threat | 0.6 | 0.55 | 1378 | 1.1 | 0.28 |
| Is22q:Time:Correct:Threat | -0.13 | 0.053 | 1378 | -2.4 | 0.015 |
|  |  |  |  |  |  |
|  |  |  |  |  |  |
|  |  | Random Effects | |  |  |
| Coefficient Name | lower | est. | upper |  |  |
| Intercept | 0.33729218 | 0.49424533 | 0.72423394 |  |  |
| Time | 0.03247815 | 0.04732734 | 0.06896565 |  |  |

| **PC6** |  | Fixed Effects | |  |  |
| --- | --- | --- | --- | --- | --- |
| Coefficient Name | Estimate | Std.Error | DF | t-value | p-value |
| (Intercept) | 1.6 | 0.83 | 1384 | 1.9 | 0.053 |
| Age (months) | -0.0014 | 8.00E-04 | 57 | -1.7 | 0.086 |
| Sex (Female) | -0.094 | 0.072 | 57 | -1.3 | 0.19 |
| Total Brain Volume | -5.20E-07 | 3.40E-07 | 57 | -1.5 | 0.13 |
| Mean Head Motion | 0.36 | 0.4 | 57 | 0.89 | 0.38 |
| Handedness | 0.096 | 0.078 | 57 | 1.2 | 0.22 |
| 22q Status | -0.077 | 0.089 | 57 | -0.87 | 0.39 |
| Time | -0.43 | 0.65 | 1384 | -0.66 | 0.51 |
| Time^2 | -0.099 | 0.21 | 1384 | -0.47 | 0.64 |
| Time^3 | 0.042 | 0.029 | 1384 | 1.5 | 0.14 |
| Time^4 | -0.0039 | 0.0017 | 1384 | -2.2 | 0.026 |
| Time^5 | 0.00011 | 3.80E-05 | 1384 | 2.8 | 0.0059 |
| Threat | -0.18 | 0.18 | 1384 | -1 | 0.31 |
| Time:Threat | 0.11 | 0.048 | 1384 | 2.3 | 0.019 |
| Time^2:Threat | -0.0063 | 0.0026 | 1384 | -2.5 | 0.014 |
|  |  |  |  |  |  |
|  |  |  |  |  |  |
|  |  | Random Effects | |  |  |
| Coefficient Name | lower | est. | upper |  |  |
| Intercept | 0.56030008 | 0.780413 | 1.08699691 |  |  |
| Time | 0.1786622 | 0.23806481 | 0.31721794 |  |  |
| Time^2 | 0.00959408 | 0.01278343 | 0.01703302 |  |  |
