# Supplementary material for: Altered functional brain dynamics in chromosome 22q11.2 deletion syndrome during facial affect processing
Source: Mol Psychiatry. 2021 Oct 22;27(2):1158–66. doi: 10.1038/s41380-021-01302-y (PMC9023602; doi:10.1038/s41380-021-01302-y)
Supplement: Supplementary file 2 — Supplement [file 41380_2021_1302_MOESM2_ESM.pdf]

Altered functional brain dynamics in chromosome 22q11.2 deletion syndrome during facial affect processing

Eli J. Cornblath[1,2], Arun Mahadevan[2], Xiaosong He[2], Kosha Ruparel[3], David M. Lydon-Staley[2,8], Tyler M. Moore[3], Ruben C. Gur[3,6,9], Elaine H. Zackai[10], Beverly Emanuel[11], Donna M. McDonald-McGinn[10], Daniel H. Wolf[3], Theodore D. Satterthwaite[3], David R. Roalf[3], Raquel E. Gur[3,6,9,†], and Danielle S. Bassett[2,3,5,6,7,12,13,†]

[1]*Department of Neuroscience, Perelman School of Medicine,*
[2]*Department of Bioengineering, School of Engineering & Applied Science,*
[3]*Department of Psychiatry, Perelman School of Medicine,*
[4]*Department of Biostatistics, Epidemiology, & Informatics, Perelman School of Medicine,*
[5]*Department of Physics & Astronomy, College of Arts & Sciences,*
[6]*Department of Neurology, Perelman School of Medicine,*
[7]*Department of Electrical & Systems Engineering, School of Engineering & Applied Science,*
[8]*Annenberg School for Communication, College of Arts & Sciences,*
[9]*Department of Radiology, Perelman School of Medicine,*
[10]*22q and You and Clinical Genetics Centers, Children's Hospital of Philadelphia,*
[11]*Division of Human Genetics, Children's Hospital of Philadelphia,*
Department of Pediatrics, Perelman School of Medicine,
University of Pennsylvania, Philadelphia, PA 19104, USA
[12]*Santa Fe Institute, Santa Fe, NM 87501, USA*
[13]*To whom correspondence should be addressed: dsb@seas.upenn.edu and*
[†] *These authors contributed equally.*

SUPPLEMENTARY INFORMATION

Image acquisition

MRI data were acquired on a 3 Tesla Siemens Tim Trio whole-body scanner and 32-channel head coil at the Hospital of the University of Pennsylvania. High-resolution T1-weighted images (TR = 1810 ms, TE = 3.51 ms, FOV = 180 × 240 mm, matrix = 256 × 192, 160 slices, TI = 1100 ms, flip angle = 9 degrees, effective voxel resolution of 0.9375 × 0.9375 × 1mm) were acquired for each subject. All subjects underwent functional imaging (TR = 3000 ms; TE = 32 ms; flip angle = 90 degrees; FOV = 192 × 192 mm; matrix = 64 × 64; slices = 46; slice thickness = 3 mm; slice gap = 0 mm; effective voxel resolution = 3.0 × 3.0 × 3.0 mm) during the emotion-identification task sequence[1]. Throughout the study, subjects' heads were stabilized in the head coil using one foam pad over each ear and a third pad over the top of the head in order to minimize motion. The emotion identification task was displayed using Presentation software, and both responses and response times were recorded using a custom fiberoptic response pad. Prior to any image acquisition, subjects were acclimated to the MRI environment via a mock scanning session in a decommissioned scanner. Mock scanning was accompanied by acoustic recordings of gradient coil noise produced by each scanning pulse sequence. Feedback regarding head motion was provided using the MoTrack motion tracking system (Psychology Software Tools, Inc., Sharpsburg, PA).

Image processing

Results included in this manuscript come from preprocessing performed using *fMRIPrep* 1.5.8[2,3] (RRID:SCR_016216), which is based on *Nipype* 1.4.1[4,5] (RRID:SCR_002502).

Anatomical data preprocessing using fMRIprep software

The T1-weighted (T1w) image was corrected for intensity non-uniformity (INU) with `N4BiasFieldCorrection`[6], distributed with ANTs 2.2.0[7] (RRID:SCR_004757), and used as the T1w-reference throughout the workflow. The T1w-reference was then skull-stripped with a *Nipype* implementation of the `antsBrainExtraction.sh` workflow (from

ANTs), using OASIS30ANTs as target template. Brain tissue segmentation of cerebrospinal fluid (CSF), white-matter (WM) and gray-matter (GM) was performed on the brain-extracted T1w using `fast`[8]. Brain surfaces were reconstructed using `recon-all`[9], and the brain mask estimated previously was refined with a custom variation of the method to reconcile ANTs-derived and FreeSurfer-derived segmentations of the cortical gray-matter of Mindboggle[10]. Volume-based spatial normalization to one standard space (MNI152NLin2009cAsym) was performed through nonlinear registration with `antsRegistration` (ANTs 2.2.0), using brain-extracted versions of both T1w reference and the T1w template. The following template was selected for spatial normalization: *ICBM 152 Nonlinear Asymmetrical template version 2009c* [[11], RRID:SCR_008796; TemplateFlow ID: MNI152NLin2009cAsym].

Functional data preprocessing using fMRIprep software

For each BOLD run found per subject (across all tasks and sessions), the following preprocessing was performed. First, a reference volume and its skull-stripped version were generated using a custom methodology of *fMRIPrep*. Susceptibility distortion correction (SDC) was omitted. The BOLD reference was then co-registered to the T1w reference using `bbregister` (FreeSurfer) which implements boundary-based registration[12]. Co-registration was configured with six degrees of freedom. Head-motion parameters with respect to the BOLD reference (transformation matrices, and six corresponding rotation and translation parameters) are estimated before any spatiotemporal filtering using MCFLIRT `Jenkinson2002`[13]. BOLD runs were slice-time corrected using `3dTshift` from AFNI 20160207[14] (RRID:SCR_005927). The BOLD time-series were resampled to surfaces on the following spaces: *fsaverage5*. The BOLD time-series (including slice-timing correction when applied) were resampled into their original, native space by applying the transforms to correct for head-motion. These resampled BOLD time-series will be referred to as *preprocessed BOLD in original space*, or just *preprocessed BOLD*. The BOLD time-series were resampled into standard space, generating a *preprocessed BOLD run in ['MNI152NLin2009cAsym'] space*. First, a reference volume and its skull-stripped version were generated using a custom methodology of *fMRIPrep*. Several confounding time-series were calculated based on the *preprocessed BOLD*: framewise displacement (FD), DVARS, and three region-wise global signals. FD and DVARS are calculated for each functional run, using their implementations in *Nipype*[15]. The three global signals are extracted within the CSF, the WM, and the whole-brain masks. Additionally, a set of physiological regressors were extracted to allow for component-based noise correction[16]. Principal components are estimated after high-pass filtering the *preprocessed BOLD* time-series (using a discrete cosine filter with 128s cut-off) for the two *CompCor* variants: temporal (tCompCor) and anatomical (aCompCor). tCompCor components are then calculated from the top 5% variable voxels within a mask covering the subcortical regions. This subcortical mask is obtained by heavily eroding the brain mask, which ensures it does not include cortical GM regions. For aCompCor, components are calculated within the intersection of the aforementioned mask and the union of CSF and WM masks calculated in T1w space, after their projection to the native space of each functional run (using the inverse BOLD-to-T1w transformation). Components are also calculated separately within the WM and CSF masks. For each CompCor decomposition, the k components with the largest singular values are retained, such that the retained components' time series are sufficient to explain 50 percent of variance across the nuisance mask (CSF, WM, combined, or temporal). The remaining components are dropped from consideration. The head-motion estimates calculated in the correction step were also placed within the corresponding confounds file. The confound time series derived from head motion estimates and global signals were expanded with the inclusion of temporal derivatives and quadratic terms for each[17]. Frames that exceeded a threshold of 0.5 mm FD or 1.5 standardised DVARS were annotated as motion outliers. All resamplings can be performed with *a single interpolation step* by composing all the pertinent transformations (i.e. head-motion transform matrices, susceptibility distortion correction when available, and co-registrations to anatomical and output spaces). Gridded (volumetric) resamplings were performed using `antsApplyTransforms` (ANTs), configured with Lanczos interpolation to minimize the smoothing effects of other kernels[18]. Non-gridded (surface) resamplings were performed using `mri_vol2surf` (FreeSurfer).

Many internal operations of *fMRIPrep* use *Nilearn* 0.6.1[19] (RRID:SCR_001362), mostly within the functional processing workflow. For more details of the pipeline, see the section corresponding to workflows in *fMRIPrep*'s documentation.

Constrained principal component analysis of emotion identification task data

In order to perform constrained principal component analysis (CPCA)[20–22], we began with $(N * T) \times P$ matrix $\mathbf{X}$, containing the BOLD time series for $P = 214$ cortical and subcortical parcels (see "Functional data processing using XCP software") over $T = 204$ image acquisitions, concatenated across $N = 116$ total subjects from both HC and 22q11.2DS cohorts, such that $\mathbf{X}$ was 23664×214. Next, we constructed an FIR basis set $\mathbf{F}$ that modeled the $r = 6$

image acquisitions following the $v = 6$ task events (unique combinations of correct, incorrect, and non-responses to threatening or non-threatening stimuli[23,24]) for each of the N subjects as a binary indicator, plus an intercept term. Initially, $\mathbf{F}$ is an $(N * T) \times (1 + (r * v * N))$ matrix (here, 23664×4177). Some subjects were either missing task response data or had a non-response rate of $> 30\%$, so rows of BOLD data in $\mathbf{X}$ and columns of regressors in $\mathbf{F}$ were removed. Additionally, some subjects did not have data for a particular response (i.e. a subject identified all stimuli correctly), so we could not construct regressors for them. After these two exclusions, $\mathbf{X}$ was 21199×214 and $\mathbf{F}$ was 21199×3065. To implement this method while preserving the link between each subject's responses and their BOLD time series, we operated only on the 21199 non-missing elements of the 23664-row matrix while keeping their original positions in place. Thus, for simplicity and consistency with our implementation, we will describe all matrices in their original size. Moving forward to the first step of the CPCA procedure for extracting task-related signals, we fit the regression equation

$$\mathbf{X} = \mathbf{B}\mathbf{F} + \mathbf{E}_1, \tag{1}$$

where $\mathbf{B}$ is a $(1 + (r * v * N)) \times P$ matrix of regression weights from the fitted model, and $\mathbf{E}_1$ is an $(N * T) \times P$ matrix of error terms. The fitted values of Equation 1 contain the variance in $\mathbf{X}$ that can be explained by the FIR basis set $\mathbf{F}$ and can be described as

$$\hat{\mathbf{X}}_t = \mathbf{B}\mathbf{F}. \tag{2}$$

Next, we decompose the task-related variance in $\hat{\mathbf{X}}_t$ using PCA, following the equation

$$\hat{\mathbf{X}}_t = \mathbf{U}\mathbf{D}\mathbf{V}^\intercal, \tag{3}$$

where $\mathbf{U}$ is an $(N * T) \times (N * T)$ matrix, $\mathbf{D}$ is an $(N * T) \times P$ diagonal matrix of singular values associated with each component, and $\mathbf{V}$ is a $P \times P$ matrix whose columns contain orthonormal spatial weights on brain regions for each component. We obtain $\mathbf{Y}$, the $(N * T) \times P$ matrix of temporal weights of each component at each modeled BOLD time point, by projecting $\hat{\mathbf{X}}_t$ into the space defined by $\mathbf{V}$, as described by the equation

$$\mathbf{Y} = \hat{\mathbf{X}}_t\mathbf{V}. \tag{4}$$

Finally, in order to relate the temporal weights of each PC to the task events, we perform a second regression step by fitting the equation

$$\mathbf{Y} = \mathbf{M}\mathbf{F} + \mathbf{E}_2, \tag{5}$$

where $\mathbf{M}$ is a $(1 + (r * v * N)) \times P$ matrix of regression weights from the fitted model, and $\mathbf{E}_2$ is an $(N * T) \times P$ matrix of error terms. $\mathbf{M}$ contains the estimated temporal response of each of the P principal components to each of the v task events for each of the N subjects.

Bootstrapping analysis of CPCA components

In order to facilitate the interpretation of these task-evoked modes of brain activity and utilize them to understand potential alterations in brain dynamics in 22q11.2DS, we performed two critical quality control analyses. First, we obtained distributions of each element of the spatial loadings by repeating the entire CPCA procedure using 10,000 bootstrapped samples of the entire dataset. We used these empiric confidence intervals to compute a two-tailed p-value for each element of the first 6 spatial loadings, and subsequently thresholded the spatial maps at $p < 10^{-4}$ (Fig. 2a for PCs 1-5, and Fig. S1) for the purposes of determining the extent of positive or negative spatial loading in each component not attributable to sampling error.

Second, because we sought to compare temporal expression of group-defined spatial modes of activity, it was important to ensure that group spatial differences did not underlie group temporal differences. To test for this possibility, we performed the CPCA procedure on each group separately and computed the variance explained in bootstrapped samples of each cohort's BOLD signal by the principal axes of the opposite group, as well as by the group solution. We found that both the group-derived spatial components and the cohort-specific spatial component explained similar amounts of variance in each cohort's BOLD data, suggesting that aggregating the two groups to identify a common set of axes is an appropriate approach (Fig. S3a). For each component, the dark blue bar and dark red bar are approximately the same height, indicating that over many bootstrapped samples, a similar amount of variance is explained in 22q11.2DS BOLD data by components obtained from the full sample as is explained by components obtained from individuals with 22q11.2DS only. Similarly, the light blue and light green bars are

approximately the same height for each component, indicating that over many bootstrapped samples, a similar amount of variance is explained in HC BOLD data by components obtained from the full sample as is explained by components from HCs only. Note that different amounts of variance can be explained by the group solution in each group due to variable temporal expression of the same spatial component, without necessitating that the model is a poor fit for one group or the other. Overall, these results suggest that the group PCA model fits each cohort as well as each cohort's PCA model fits its own data.

Multilevel growth model selection procedure

After obtaining the matrix $\mathbf{M}$ of the temporal responses of each principal component to each stimulus, we next sought to describe the trajectory of PC activity across time. In doing so, we also sought to identify differences in these trajectories between HCs and individuals with 22q11.2DS, and across the different task events (each of the 4 combinations of correct and incorrect responses to threat and non-threat stimuli). First, we discarded the rows of $\mathbf{M}$ that corresponded to non-response trials, because while it was important to make sure these trials did not go unmodeled in the CPCA procedure, we did not expect non-response trials to reveal consistent patterns of event-related brain activity across subjects. We also discarded column 1 (corresponding to global signal, shown in Fig. S2) and columns 7 through P of $\mathbf{M}$ in order to model only the first 5 principal components, as determined by the scree plot (Fig. S1a). Next, we carried out a model selection procedure to accurately and parsimoniously model the trajectory of each PC's activation across time and its moderation by 22q11.2DS status and response type, while controlling for age, sex, total brain volume, mean framewise displacement during task scans, and handedness[25]. In each step of the procedure, we used full maximum likelihood estimation to fit a series of multilevel growth models with the `nlme`[26] package in R, where the dependent variable is always the estimated response of the ith principal component score, contained in the ith column of $\mathbf{M}$, and the independent variables were determined by the model selection procedure. We specified a two-level model, where repeated measures of activity following each task event were nested within each participant.

In the model selection procedure, we followed the approach taken by a previous study[25] to sequentially add predictors while ensuring that the increase in model complexity provided a statistically significant improvement in model fit. In each step, a model with fewer parameters was compared to a model with more parameters. In order to select the more complex model as the new "gold standard," the more complex model had to satisfy 3 criteria:

1. Lower value of the Akaike Information Criteria (AIC)[27]

2. $p < 0.05$ for the log-likelihood ratio test, to assess whether the log-likelihood of the more complex model is greater than the more simple model

3. $p < 0.05$ for the additional coefficients in the more complex model

Using these criteria for determining the superiority of one model over another, we employed the following standardized, partially supervised model selection procedure, annotated with the functions used to implement them in our publicly available code repository (`code/statfxns/lme_msfxns.R`):

1. *Fit base model* (`lme.ms`) : Fit a model Φ_o with fixed effects for age, sex, total brain volume, mean framewise displacement during task scans, handedness, and group membership (22q11.2DS or control), and random intercepts for subject, as defined by the equation:

$$\mathbf{M}_i = \mathbf{C}\beta + \mathbf{Z}\mathbf{b} + \epsilon, \tag{6}$$

   where $\mathbf{M}_i$ is the vector of estimated responses for the ith principal component, β is a vector of optimized regression weights for the p fixed effects for all independent variables in the matrix $\mathbf{C}$, $\mathbf{b}$ is a vector of optimized, subject-specific regression weights that model random effects for time and time polynomials contained in $\mathbf{Z}$, and ϵ is a vector of normally distributed errors. $\mathbf{b}$ is normally distributed with 0 mean, and both $\mathbf{C}\beta$ and $\mathbf{Z}\mathbf{b}$ contain intercept terms.

2. *Add fixed effects of time* (`lme.compare`) : Compare Φ_o to ξ_k, where ξ_k contains the parameters of Φ_o plus a kth order polynomial of time as a predictor. Discard all ξ_k that are inferior to Φ_o.

   (a) *Find the most appropriate model with a significant fixed effect of time* (`lme.selectbest`) : Compare the remaining models in ξ to one another and set Φ_t equal to the most superior ξ_k, which contains a polynomial of order t.

(b) *Add fixed interactions between time, stimulus type, and response type* (`lme.stepdown`) : Compare Φ_t to ξ_k, where ξ_k contains the parameters of Φ_t plus 3-way interactions between response type, stimulus type, and time polynomials from order k to order 0, where the maximum value of k is t. Set Φ_t equal to ξ_k with the largest value of k for which ξ_k was superior to Φ_t.

   i. *Add fixed interactions between time and stimulus type, or between time and response types* (`lme.stepdown`) : If there are time polynomial terms without 3-way interactions, compare Φ_t to ξ_k, where ξ_k contains the parameters of Φ_t plus 2-way interactions between response type or stimulus type (sequentially) and time polynomials from order k to order 0, where the maximum value of k is t. Set Φ_t equal to ξ_k with the largest value of k for which ξ_k was superior to Φ_t.

(c) *Add random effects of time* (`lme.stepup`) : Compare Φ_t to ξ_k, where ξ_k contains the parameters of Φ_t plus random effects of time from order 1 to order k, where the maximum value of k is t. Set Φ_t equal to ξ_k with the largest value of k for which ξ_k was superior to Φ_t.

(d) *Add interactions between 22q11.2DS status and time* (`lme.selectbest`) : Compare Φ_t to ξ_k, where ξ_k contains the parameters of Φ_t plus interactions between group membership and time polynomials from order 1 to k, where the maximum value of k is t. Set Φ_t equal to the most superior ξ_k.

(e) *Add interactions between 22q11.2DS status, time, stimulus type, and response type* (`lme.stepdown`) : Compare Φ_t to ξ_k, where ξ_k contains the parameters of Φ_t plus 4-way interactions between response type, stimulus type, group membership, and time polynomials from order k to order 0, where the maximum value of k is t. Set Φ_t equal to ξ_k with the largest value of k for which ξ_k was superior to Φ_t.

   i. *Add interactions between 22q11.2DS status, stimulus type, and time, or between 22q11.2DS status, response type, and time* (`lme.stepdown`) : If there are time polynomial terms without 4-way interactions, compare Φ_t to ξ_k, where ξ_k contains the parameters of Φ_t plus 3-way interactions between response type or stimulus type (sequentially), group membership, and time polynomials from order k to order 0, where the maximum value of k is t. Set Φ_t equal to ξ_k with the largest value of k for which ξ_k was superior to Φ_t.

In the above procedure, we began with covariates in the model in case these confounding factors may have obscured a relationship with time. The `lme.selectbest` function was used instead of the `lme.stepdown` or `lme.stepup` functions when it was possible for a model differing by more than one parameter to outperform Φ_o or Φ_t. This situation occurs when comparing polynomials of time, where it is possible for a linear time model to show equivalent performance with a 0th order no-time model, but a quadratic or higher order model can outperform the no-time model due to the non-linear relationship with time. The coefficient table of the final model for $\mathbf{M}_i$ was used to assess the relationships between 22q11.2DS group membership, time, stimulus type, and response type. These tables are attached as Supplementary Data File 1.

[1] Satterthwaite TD, Elliott MA, Ruparel K, Loughead J, Prabhakaran K, Calkins ME et al. Neuroimaging of the Philadelphia Neurodevelopmental Cohort. *NeuroImage* 86:544–553, 2014. ISSN 10959572. doi:10.1016/j.neuroimage.2013.07.064.

[2] Esteban O, Markiewicz CJ, Blair RW, Moodie CA, Isik AI, Erramuzpe A et al. fMRIPrep: a robust preprocessing pipeline for functional MRI. *Nature methods* 16(1):111–116, 2019.

[3] Esteban O, Blair R, Markiewicz CJ, Berleant SL, Moodie C, Ma F et al. fMRIPrep. *Software* 2018. doi: 10.5281/zenodo.852659.

[4] Gorgolewski K, Burns CD, Madison C, Clark D, Halchenko YO, Waskom ML et al. Nipype: a flexible, lightweight and extensible neuroimaging data processing framework in Python. *Frontiers in Neuroinformatics* 5:13, 2011. doi: 10.3389/fninf.2011.00013.

[5] Gorgolewski KJ, Esteban O, Markiewicz CJ, Ziegler E, Ellis DG, Notter MP et al. Nipype. *Software* 2018. doi: 10.5281/zenodo.596855.

[6] Tustison NJ, Avants BB, Cook PA, Zheng Y, Egan A, Yushkevich PA et al. N4ITK: Improved N3 Bias Correction. *IEEE Transactions on Medical Imaging* 29(6):1310–1320, 2010. ISSN 0278-0062. doi:10.1109/TMI.2010.2046908.

[7] Avants BB, Epstein CL, Grossman M, and Gee JC. Symmetric diffeomorphic image registration with cross-correlation: Evaluating automated labeling of elderly and neurodegenerative brain. *Medical Image Analysis* 12(1):26–41, 2008. ISSN 1361-8415. doi:10.1016/j.media.2007.06.004.

[8] Zhang Y, Brady M, and Smith S. Segmentation of brain MR images through a hidden Markov random field model and the expectation-maximization algorithm. *IEEE Transactions on Medical Imaging* 20(1):45–57, 2001. ISSN 0278-0062. doi:10.1109/42.906424.

[9] Dale AM, Fischl B, and Sereno MI. Cortical Surface-Based Analysis: I. Segmentation and Surface Reconstruction. *NeuroImage* 9(2):179–194, 1999. ISSN 1053-8119. doi:10.1006/nimg.1998.0395.

[10] Klein A, Ghosh SS, Bao FS, Giard J, Häme Y, Stavsky E et al. Mindboggling morphometry of human brains. *PLOS Computational Biology* 13(2):e1005350, 2017. ISSN 1553-7358. doi:10.1371/journal.pcbi.1005350.

[11] Fonov VS, Evans AC, McKinstry RC, Almli CR, and Collins DL. Unbiased nonlinear average age-appropriate brain templates from birth to adulthood. *NeuroImage* 47, Supple:S102, 2009. doi:10.1016/S1053-8119(09)70884-5.

[12] Greve DN and Fischl B. Accurate and robust brain image alignment using boundary-based registration. *NeuroImage* 48(1):63–72, 2009. ISSN 1095-9572. doi:10.1016/j.neuroimage.2009.06.060.

[13] Jenkinson M, Bannister P, Brady M, and Smith S. Improved optimization for the robust and accurate linear registration and motion correction of brain images. *NeuroImage* 17(2):825–41, 2002. ISSN 1053-8119.

[14] Cox RW and Hyde JS. Software tools for analysis and visualization of fMRI data. *NMR in Biomedicine* 10(4-5):171–178, 1997. doi:10.1002/(SICI)1099-1492(199706/08)10:4/5¡171::AID-NBM453¿3.0.CO;2-L.

[15] Power JD, Mitra A, Laumann TO, Snyder AZ, Schlaggar BL, and Petersen SE. Methods to detect, characterize, and remove motion artifact in resting state fMRI. *NeuroImage* 84(Supplement C):320–341, 2014. ISSN 1053-8119. doi: 10.1016/j.neuroimage.2013.08.048.

[16] Behzadi Y, Restom K, Liau J, and Liu TT. A component based noise correction method (CompCor) for BOLD and perfusion based fMRI. *NeuroImage* 37(1):90–101, 2007. ISSN 1053-8119. doi:10.1016/j.neuroimage.2007.04.042.

[17] Satterthwaite TD, Elliott MA, Gerraty RT, Ruparel K, Loughead J, Calkins ME et al. An improved framework for confound regression and filtering for control of motion artifact in the preprocessing of resting-state functional connectivity data. *NeuroImage* 64(1):240–256, 2013. ISSN 10538119. doi:10.1016/j.neuroimage.2012.08.052.

[18] Lanczos C. Evaluation of Noisy Data. *Journal of the Society for Industrial and Applied Mathematics Series B Numerical Analysis* 1(1):76–85, 1964. ISSN 0887-459X. doi:10.1137/0701007.

[19] Abraham A, Pedregosa F, Eickenberg M, Gervais P, Mueller A, Kossaifi J et al. Machine learning for neuroimaging with scikit-learn. *Frontiers in Neuroinformatics* 8, 2014. ISSN 1662-5196. doi:10.3389/fninf.2014.00014.

[20] Goghari VM, Sanford N, Spilka MJ, and Woodward TS. Task-Related Functional Connectivity Analysis of Emotion Discrimination in a Family Study of Schizophrenia. *Schizophrenia Bulletin* 43(6):1348–1362, 2017. ISSN 0586-7614. doi: 10.1093/schbul/sbx004.

[21] Woodward TS, Tipper CM, Leung AL, Lavigne KM, Sanford N, and Metzak PD. Reduced functional connectivity during controlled semantic integration in schizophrenia: A multivariate approach. *Human brain mapping* 36(8):2948–2964, 2015.

[22] Sanford N, Whitman JC, and Woodward TS. Task-merging for finer separation of functional brain networks in working memory. *Cortex* 125:246–271, 2020. ISSN 19738102. doi:10.1016/j.cortex.2019.12.014.

[23] Satterthwaite TD, Wolf DH, Gur RC, Ruparel K, Valdez JN, Gur RE et al. Frontolimbic responses to emotional face memory: The neural correlates of first impressions. *Human Brain Mapping* 30(11):3748–3758, 2009. ISSN 10659471. doi:10.1002/hbm.20803.

[24] Gur RE, Loughead J, Kohler CG, Elliott MA, Lesko K, Ruparel K et al. Limbic activation associated with misidentification of fearful faces and flat affect in schizophrenia. *Archives of General Psychiatry* 64(12):1356–1366, 2007. ISSN 0003990X. doi:10.1001/archpsyc.64.12.1356.

[25] Braams BR, van Duijvenvoorde AC, Peper JS, and Crone EA. Longitudinal changes in adolescent risk-taking: A comprehensive study of neural responses to rewards, pubertal development, and risk-taking behavior. *Journal of Neuroscience* 35(18):7226–7238, 2015. ISSN 15292401. doi:10.1523/JNEUROSCI.4764-14.2015.

[26] Pinheiro J, Bates D, DebRoy S, Sarkar D, and R Core Team. *nlme: Linear and Nonlinear Mixed Effects Models*, 2019.

[27] Akaike H. A new look at the statistical model identification. *IEEE transactions on automatic control* 19(6):716–723, 1974.

[28] Liu TT, Nalci A, and Falahpour M. The global signal in fMRI: Nuisance or Information? *NeuroImage* 150:213–229, 2017. ISSN 10959572. doi:10.1016/j.neuroimage.2017.02.036.
[29] Peres-Neto PR, Jackson DA, and Somers KM. GIVING MEANINGFUL INTERPRETATION TO ORDINATION AXES: ASSESSING LOADING SIGNIFICANCE IN PRINCIPAL COMPONENT ANALYSIS. *Ecology* 84(9):2347–2363, 2003. ISSN 0012-9658. doi:10.1890/00-0634.
[30] Schaefer A, Kong R, Gordon EM, Laumann TO, Zuo XN, Holmes AJ et al. Local-Global Parcellation of the Human Cerebral Cortex from Intrinsic Functional Connectivity MRI. *Cerebral Cortex* pages 1–20, 2017. ISSN 1047-3211. doi: 10.1093/cercor/bhx179.

SUPPLEMENTARY DATA FILES

1. Supplementary Data File 1. Coefficients for non-linear mixed effects models of each of the 6 principal component time courses.

SUPPLEMENTARY FIGURES

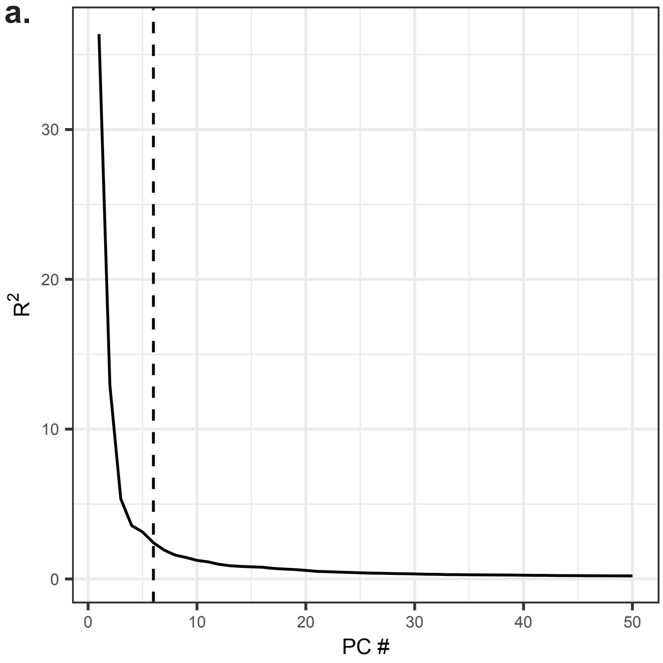

FIG. S1. **Scree plot of variance explained by PCA step in CPCA procedure.** *(a)* Scree plot showing the amount of variance explained (y-axis) by each of the first 50 principal components (x-axis).

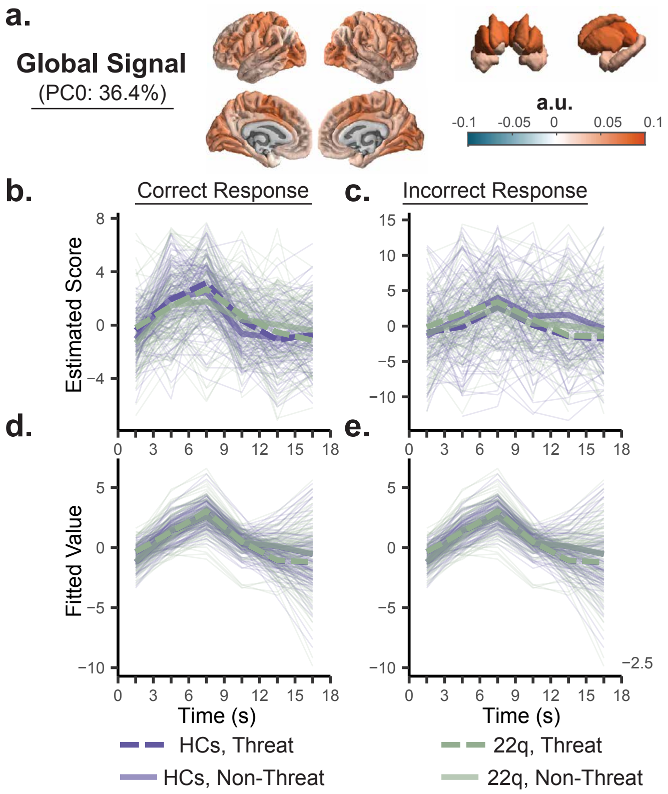

FIG. S2. **First principal component captures apparent global signal fluctuation.** *(a)* Spatial loadings of the first principal component ("PC0") of task-related variance (Fig. 1b) in emotion identification task BOLD signal reflects apparent global signal fluctuation[28]. Maps were thresholded at $p < 10^{-4}$ using bootstrap significance testing[29] and displayed on surface renderings of cortex and subcortex. *(b, c)* Mean temporal score (y-axis) of each task-evoked PC during the time (x-axis) period 0-18 seconds after correct (panel b) or incorrect (panel c) emotion identification of threatening (thick lines) and non-threatening (dashed lines) faces. The thick lines represent group average values, while the faded lines represent individual subject trajectories. *(d, e)* Multilevel growth models fit to the data in panel b (panel d) or panel c (panel e) using the partially supervised model selection procedure described in Methods and Supplementary information. These multilevel models did not contain any coefficients or interaction terms involving 22q11.2DS status.

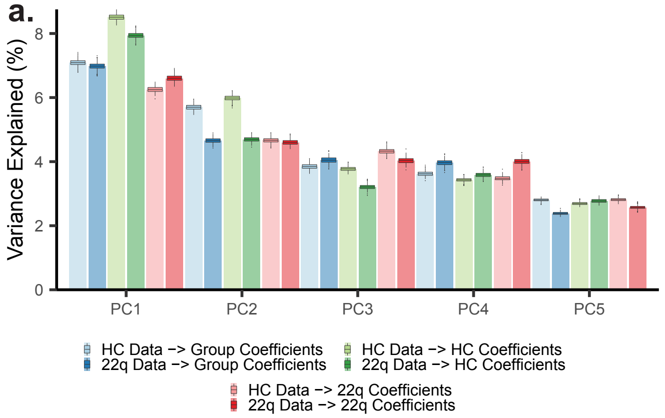

FIG. S3. **Group principal component analysis solutions capture similar amounts of variance in each cohort.** *(a)* Boxplots of explained variance (y-axis) by each component (x-axis) in either HC or 22q bootstrapped BOLD data by projecting BOLD data into a component space obtained from either HC subjects ("HC Coefficients"), 22q11.2DS subjects ("22q Coefficients"), or all subjects ("Group Coefficients"). This analysis shows that a similar amount of variance in each group is explained by components obtained from either group or the entire sample, suggesting that the group solution is sufficient to explain data from either group.

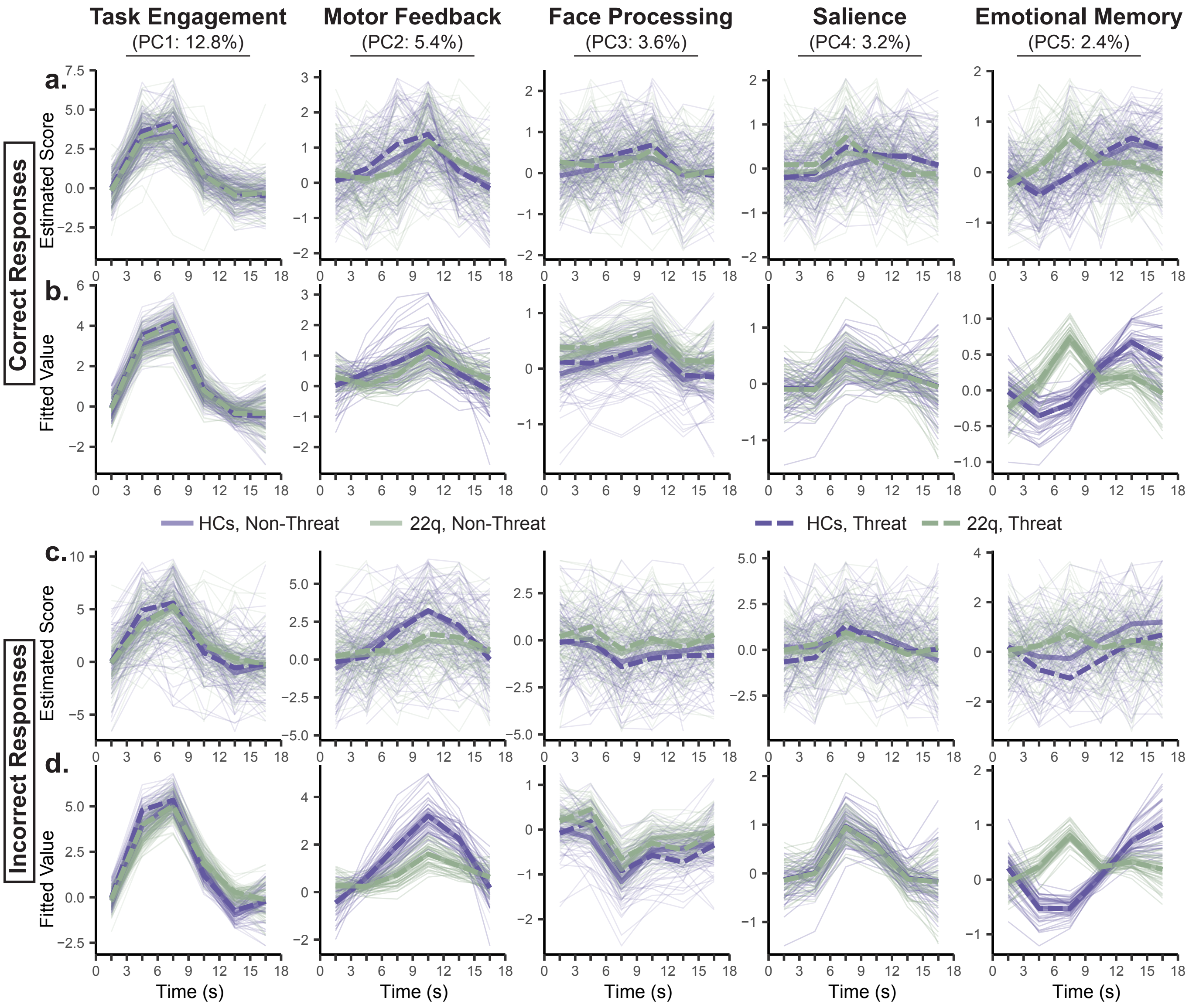

FIG. S4. **Evoked responses of CPCA components.** *(a, c)* Mean temporal score (y-axis) of each task-evoked PC during the time (x-axis) period 0-18 seconds after correct (panel a) or incorrect (panel c) emotion identification of threatening (thick lines) and non-threatening (dashed lines) faces. The thick lines represent group average values, while the faded lines represent individual subject trajectories. *(b, d)* Multilevel growth models fit to the data in panel a (panel b) or panel c (panel d), reproduced from Figure 2b,c.

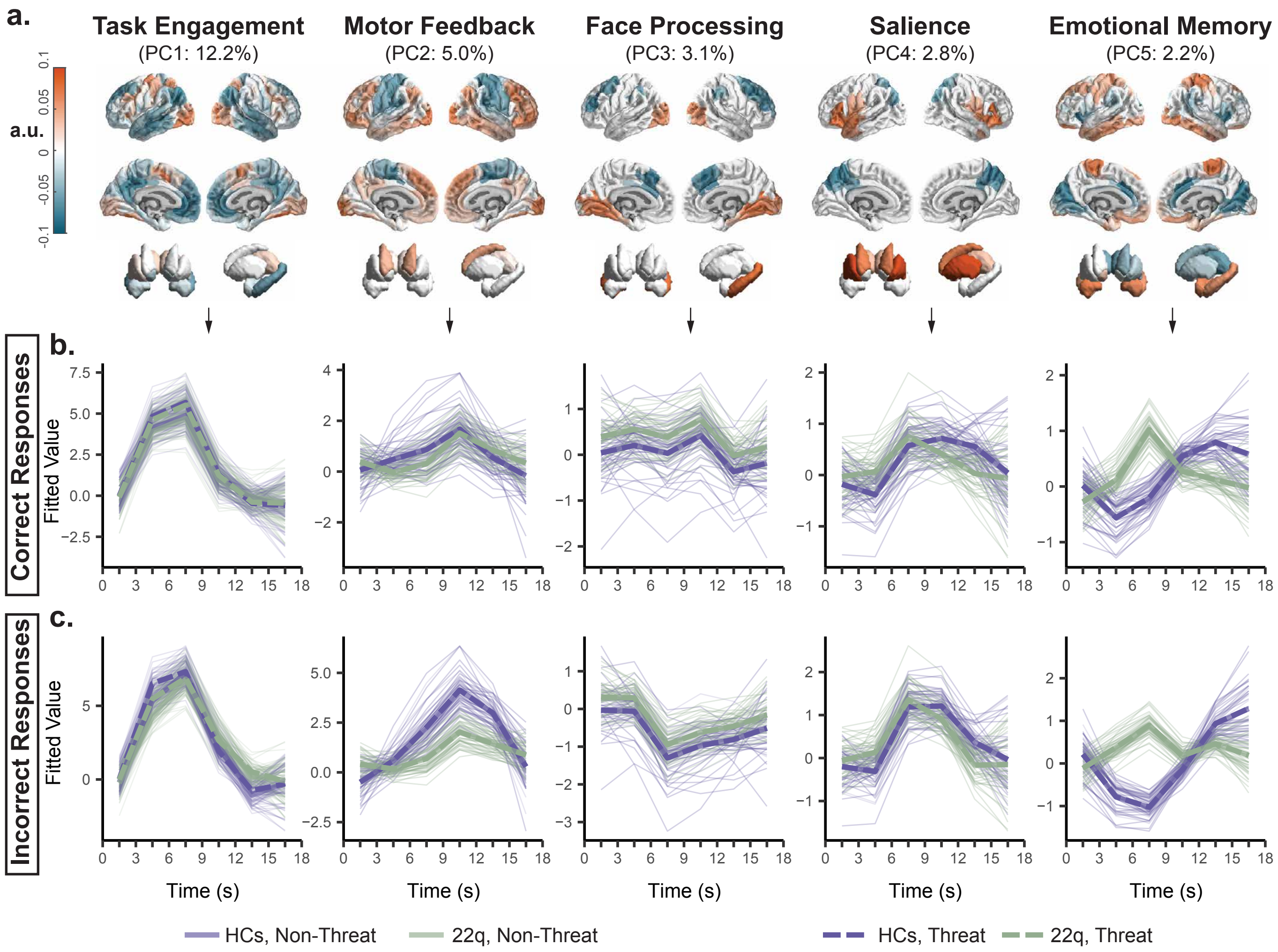

FIG. S5. **Task-evoked CPCA components using 400 node Schaefer cortical parcellation.** This figure is a replication of Fig. 2 using the 400 node Schaefer parcellation[30] with 14 subcortical nodes defined using the Harvard-Oxford atlas. *(a)* Spatial loadings of the first 5 principal components of task-related variance (Fig. 1b) in emotion identification task BOLD signal thresholded at $p < 10^{-4}$ using bootstrap significance testing[29], shown on surface renderings of cortex and subcortex. Components are named based upon the authors' interpretation of the data and existing literature on localization of brain function (see Discussion). *(b, c)* Multilevel growth models fit to the temporal scores (y-axis) of each task-evoked PC during the time (x-axis) occurring 0-18 seconds after correct (panel b) or incorrect (panel c) emotion identification of threatening (thick lines) and non-threatening (dashed lines) faces. We used a model selection procedure (see Methods) to predict each PC's scores over time from polynomials of time, stimulus type (threat or non-threat), response type (correct or incorrect), 22q status, and interactions between those variables while controlling for age, sex, total brain volume, head motion, and handedness. The best model selected through this process was used to obtain fitted values (y-axis) to describe the trajectory of each PC's score for the prototypical individual in each group (thick, opaque lines) and for each participant (thin, faded lines).

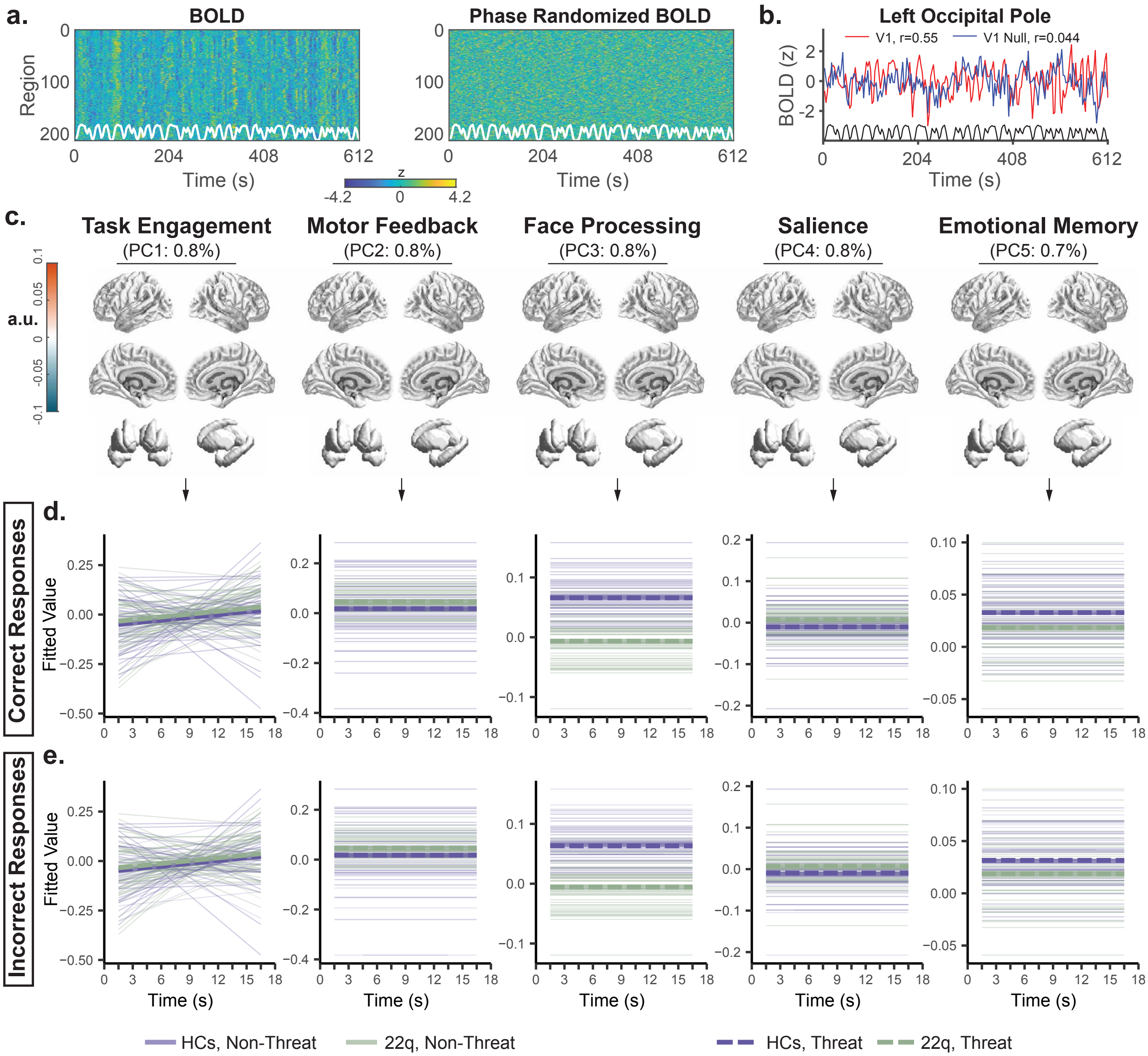

FIG. S6. **Task-evoked CPCA components with phase randomized BOLD data.** This figure is a replication of Fig. 2 with each region's BOLD time series independently phase randomized within each subject. *(a)* Original BOLD time series (left) next to phase randomized time series (right) for a selected subject. White overlay is the stimulus convolved with canonical hemodynamic response function. *(b)* BOLD time series (red) and phase randomized time series (blue) for left primary visual cortex parcel for selected subject plotted against convolved stimulus (black). Pearson correlations show weaker relationship between stimulus and signal in phase randomized data. *(c)* Spatial loadings of the first 5 principal components of task-related variance (Fig. 1b) in emotion identification task BOLD signal thresholded at $p < 10^{-4}$ using bootstrap significance testing[29], shown on surface renderings of cortex and subcortex. Components are named based upon the authors' interpretation of the data and existing literature on localization of brain function (see Discussion). *(d, e)* Multilevel growth models fit to the temporal scores (y-axis) of each task-evoked PC during the time (x-axis) occurring 0-18 seconds after correct (panel d) or incorrect (panel e) emotion identification of threatening (thick lines) and non-threatening (dashed lines) faces. We used a model selection procedure (see Methods) to predict each PC's scores over time from polynomials of time, stimulus type (threat or non-threat), response type (correct or incorrect), 22q status, and interactions between those variables while controlling for age, sex, total brain volume, head motion, and handedness. The best model selected through this process was used to obtain fitted values (y-axis) to describe the trajectory of each PC's score for the prototypical individual in each group (thick, opaque lines) and for each participant (thin, faded lines).

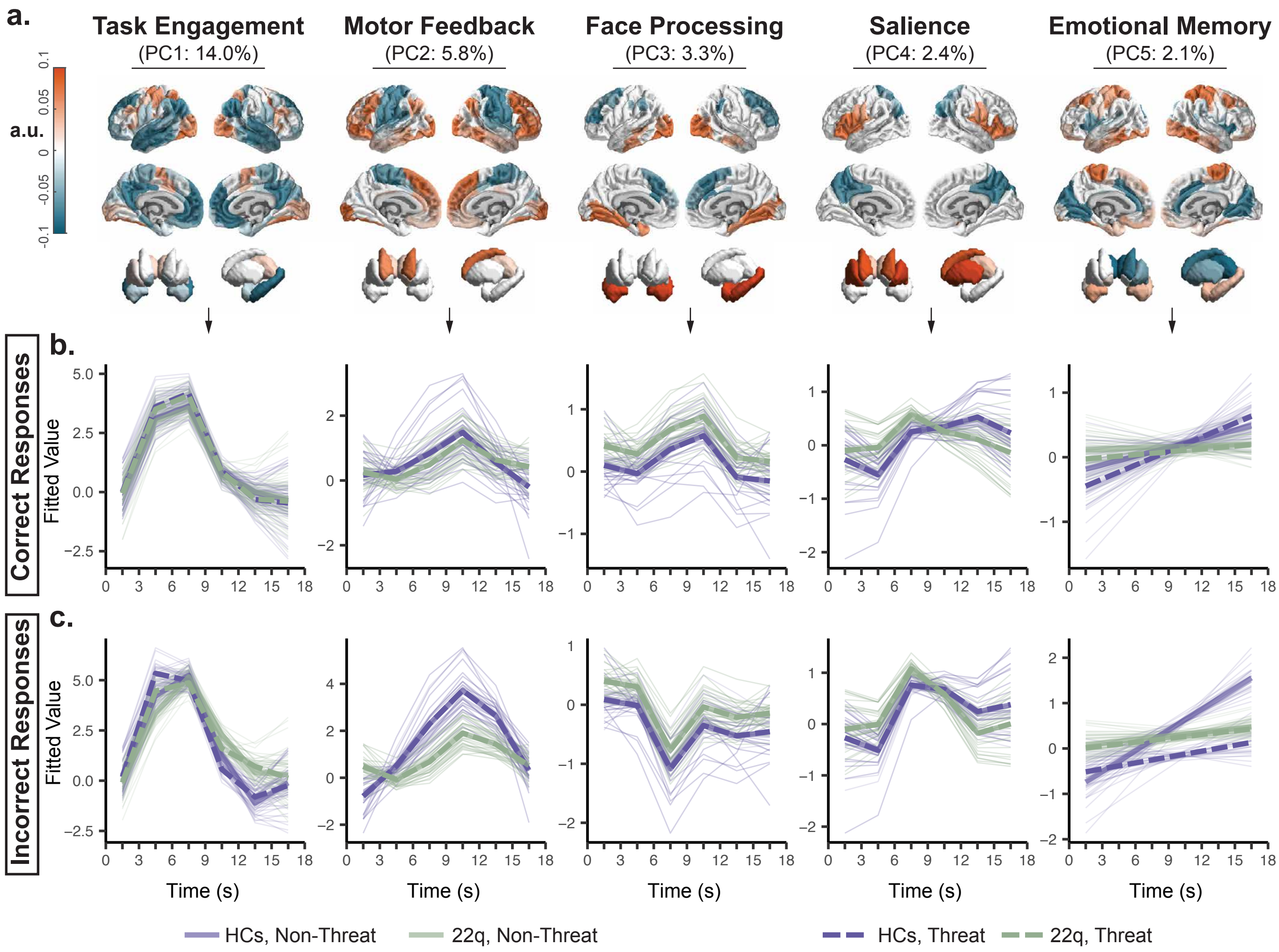

FIG. S7. **Task-evoked CPCA components using subjects with ≥75% accuracy.** This figure is a replication of Fig. 2 using 64 subjects (32 matched pairs) with ≥75% overall emotion identification accuracy. *(a)* Spatial loadings of the first 5 principal components of task-related variance (Fig. 1b) in emotion identification task BOLD signal thresholded at $p < 10^{-4}$ using bootstrap significance testing[29], shown on surface renderings of cortex and subcortex. Components are named based upon the authors' interpretation of the data and existing literature on localization of brain function (see Discussion). *(b, c)* Multilevel growth models fit to the temporal scores (y-axis) of each task-evoked PC during the time (x-axis) occurring 0-18 seconds after correct (panel *b*) or incorrect (panel *c*) emotion identification of threatening (thick lines) and non-threatening (dashed lines) faces. We used a model selection procedure (see Methods) to predict each PC's scores over time from polynomials of time, stimulus type (threat or non-threat), response type (correct or incorrect), 22q status, and interactions between those variables while controlling for age, sex, total brain volume, head motion, and handedness. The best model selected through this process was used to obtain fitted values (y-axis) to describe the trajectory of each PC's score for the prototypical individual in each group (thick, opaque lines) and for each participant (thin, faded lines).

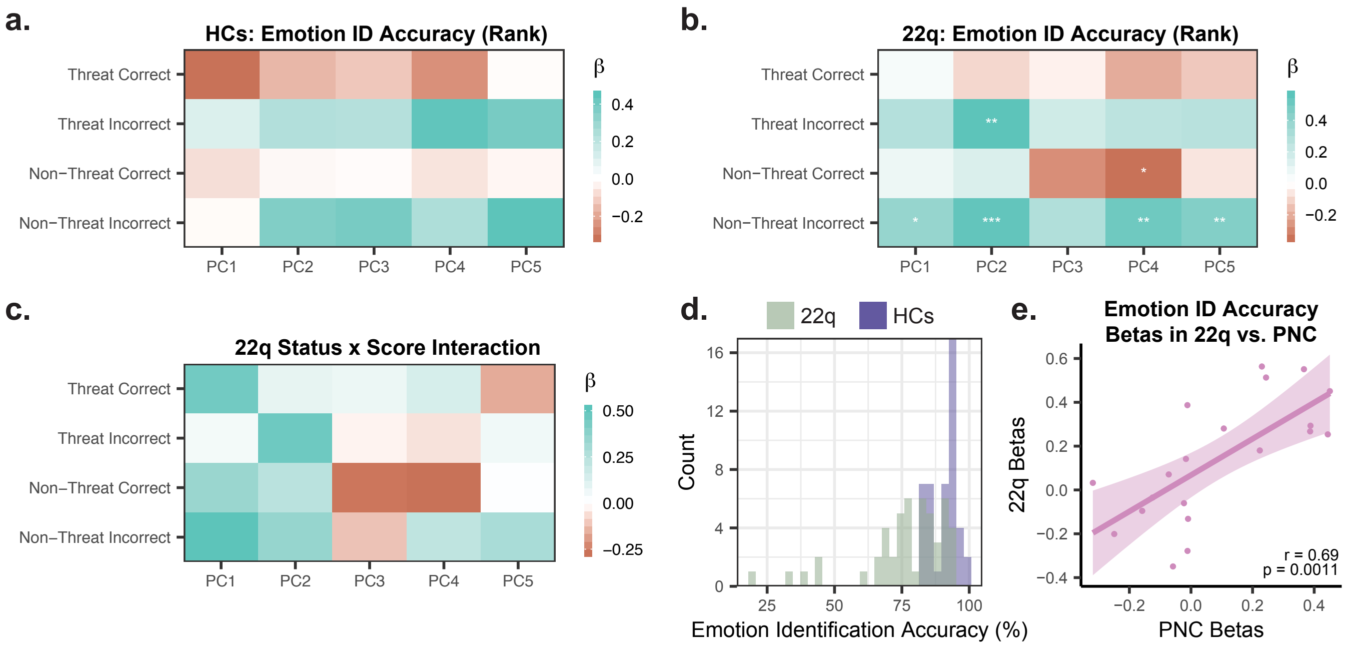

FIG. S8. **Overall task performance in healthy controls can be predicted from peak PC scores.** *(a-c)* Standardized linear regression β weights (color axis) for the peak value of each PC (x-axis) during each task event (y-axis) as a predictor of overall in-scanner emotion identification accuracy using the sample of PNC controls only (panel *a*), 22q11.2DS only (panel *b*), or the group $\times$ PC score interaction term in the full sample. All models contained age, sex, total brain volume, head motion, and handedness as covariates. Asterisks indicate level of significance after FDR correction ($q < 0.05$) over all 20 β values: *, $p_{\text{FDR}} < 0.05$. **, $p_{\text{FDR}} < 0.01$. ***, $p_{\text{FDR}} < 0.001$. *(b)* Histogram of overall emotion identification accuracy for PNC and 22q11.2DS subjects. *(c)* Linear regression β weights for PNC subjects from panel *a* (x-axis) plotted against β weights for individuals with 22q11.2DS from panel *b*.
